# Dual metabolomic profiling uncovers *Toxoplasma* manipulation of the host metabolome and the discovery of a novel parasite metabolic capability

**DOI:** 10.1371/journal.ppat.1008432

**Published:** 2020-04-07

**Authors:** William J. Olson, Bruno Martorelli Di Genova, Gina Gallego-Lopez, Anthony R. Dawson, David Stevenson, Daniel Amador-Noguez, Laura J. Knoll

**Affiliations:** 1 Department of Medical Microbiology and Immunology, University of Wisconsin—Madison, Madison, WI; 2 Morgridge Institute for Research, Madison, WI, United States of America; 3 Department of Bacteriology, University of Wisconsin—Madison, Madison, WI; University of Geneva, SWITZERLAND

## Abstract

The obligate intracellular parasite *Toxoplasma gondii* is auxotrophic for several key metabolites and must scavenge these from the host. It is unclear how *T*. *gondii* manipulates host metabolism to support its overall growth rate and non-essential metabolites. To investigate this question, we measured changes in the joint host-parasite metabolome over a time course of infection. Host and parasite transcriptomes were simultaneously generated to determine potential changes in expression of metabolic enzymes. *T*. *gondii* infection changed metabolite abundance in multiple metabolic pathways, including the tricarboxylic acid cycle, the pentose phosphate pathway, glycolysis, amino acid synthesis, and nucleotide metabolism. Our analysis indicated that changes in some pathways, such as the tricarboxylic acid cycle, were mirrored by changes in parasite transcription, while changes in others, like the pentose phosphate pathway, were paired with changes in both the host and parasite transcriptomes. Further experiments led to the discovery of a *T*. *gondii* enzyme, sedoheptulose bisphosphatase, which funnels carbon from glycolysis into the pentose phosphate pathway through an energetically driven dephosphorylation reaction. This additional route for ribose synthesis appears to resolve the conflict between the *T*. *gondii* tricarboxylic acid cycle and pentose phosphate pathway, which are both NADP+ dependent. Sedoheptulose bisphosphatase represents a novel step in *T*. *gondii* central carbon metabolism that allows *T*. *gondii* to energetically-drive ribose synthesis without using NADP+.

## Author summary

The obligate intracellular parasite *T*. *gondii* is commonly found among human populations worldwide and poses severe health risks to fetuses and individuals with AIDS. While some treatments are available they are limited in scope. A possible target for new therapies is *T*. *gondii*’s incomplete metabolism, which makes it heavily reliant on its host. In this study, we generated a joint host/parasite metabolome to better understand host manipulation by the parasite and to discover unique aspects of *T*. *gondii* metabolism that could serve as the next generation of drug targets. Metabolomic analysis of *T*. *gondii* infection over time found broad alterations to host metabolism by the parasite in both energetic and biosynthetic pathways. We discovered a new *T*. *gondii* enzyme, sedoheptulose bisphosphatase, which redirects carbon from glycolysis into the pentose phosphate pathway. The wholesale remodeling of host metabolism for optimal parasite growth is also of interest, although the mechanisms behind this host manipulation must be further studied before therapeutic targets can be identified.

## Introduction

*Toxoplasma gondii* is an obligate intracellular protozoan of the phylum Apicomplexa. It can infect any nucleated cell of any warm-blooded animal and forms a chronic infection in approximately one third of the human population [1]. Healthy human adults are often asymptomatic during infection, but *T*. *gondii* can be lethal to immunocompromised individuals and fetuses if acquired congenitally [1].

Metabolic dependencies are part of the reason that *T*. *gondii* is an obligate intracellular pathogen. *T*. *gondii* possesses a complex metabolism with most of the major metabolic pathways complete, including the tricarboxylic acid (TCA) cycle, the pentose phosphate pathway (PPP), gluconeogenesis and glycolysis [2–9]. These central pathways are generally fueled by glucose or glutamine scavenged from the host cell. Other areas of metabolism, including amino acid, nucleotide, and lipid synthesis, are partially present but rely on host metabolism to provide certain metabolites, including arginine [10], tryptophan [11], tyrosine [12], purines [13] and cholesterol [14].

Recent metabolic studies have greatly expanded our understanding of *T*. *gondii* metabolism. The *T*. *gondii* TCA cycle was found to be essential to growth along with a GABA shunt that can fuel the TCA cycle with glutamine [2]. In no glucose environments, or with glycolysis genetically ablated, *T*. *gondii* can perform glutaminolysis to provide carbon for gluconeogenesis [3,7]. This glutamine to glucose pathway relies on a gluconeogenic enzyme fructose bisphosphatase 2, which was found to be constitutively expressed and essential to growth [7]. Similarly, *T*. *gondii* catabolism of glutamine to fuel gluconeogenesis is reliant on a mitochondrial phosphoenolpyruvate carboxykinase enzyme, which is thought to play a key regulatory role in *T*. *gondii* carbon metabolism [15]. The metabolic flexibility of *T*. *gondii* is remarkable, recent work has even shown that growth will occur in environments completely lacking glucose and glutamine [16]. These and other studies are critical to expanding our understanding of *T*. *gondii* metabolism; however, the host metabolome has been largely unexplored. For other pathogens, studies that include the host metabolome have led to novel findings, including the discovery of a new metabolic gene in *Plasmodium falciparum* [17].

This current study expands the scope of *T*. *gondii* infection metabolomics to include the host metabolome. We generated a joint host-parasite metabolome over the course of infection in a human tissue culture infection model, along with matched uninfected control samples. We paired this metabolite analysis with transcriptomics of host and parasite over the same time course of infection. mRNA abundance does not give a full picture of metabolic pathway regulation, as many metabolic enzymes are heavily regulated at the translational and post translational level [18]. However, by examining host and parasite transcriptomes we were able to identify genes of interest, where a shift in metabolite abundance correlated with a change in gene expression in either the host or parasite. We found that the infection metabolome changes over the time course of infection and differs from the uninfected control in several areas. Infection changed amino acid and nucleotide synthesis, glycolysis, the TCA cycle and the PPP. Increases in the abundance of sedoheptulose-7-phosphate and sedoheptulose-1,7-bisphosphate led to the discovery of a unique parasite metabolic enzyme, sedoheptulose bisphosphatase, which does not appear to be present in mammalian host cells [19–21]. Further experiments with genetically manipulated parasites showed that *T*. *gondii* sedoheptulose bisphosphatase energetically drives carbon into the non-oxidative pentose phosphate pathway, where it can be converted into ribose. This novel activity gives *T*. *gondii* an additional pathway for synthesizing ribose and acts as a metabolic switch to redirect carbon from glycolysis into ribose synthesis. This analysis expands our understanding of host and parasite metabolism during *T*. *gondii* infection.

## Results

### The *T*. *gondii*-host joint metabolome changes throughout infection

**Fig 1:**
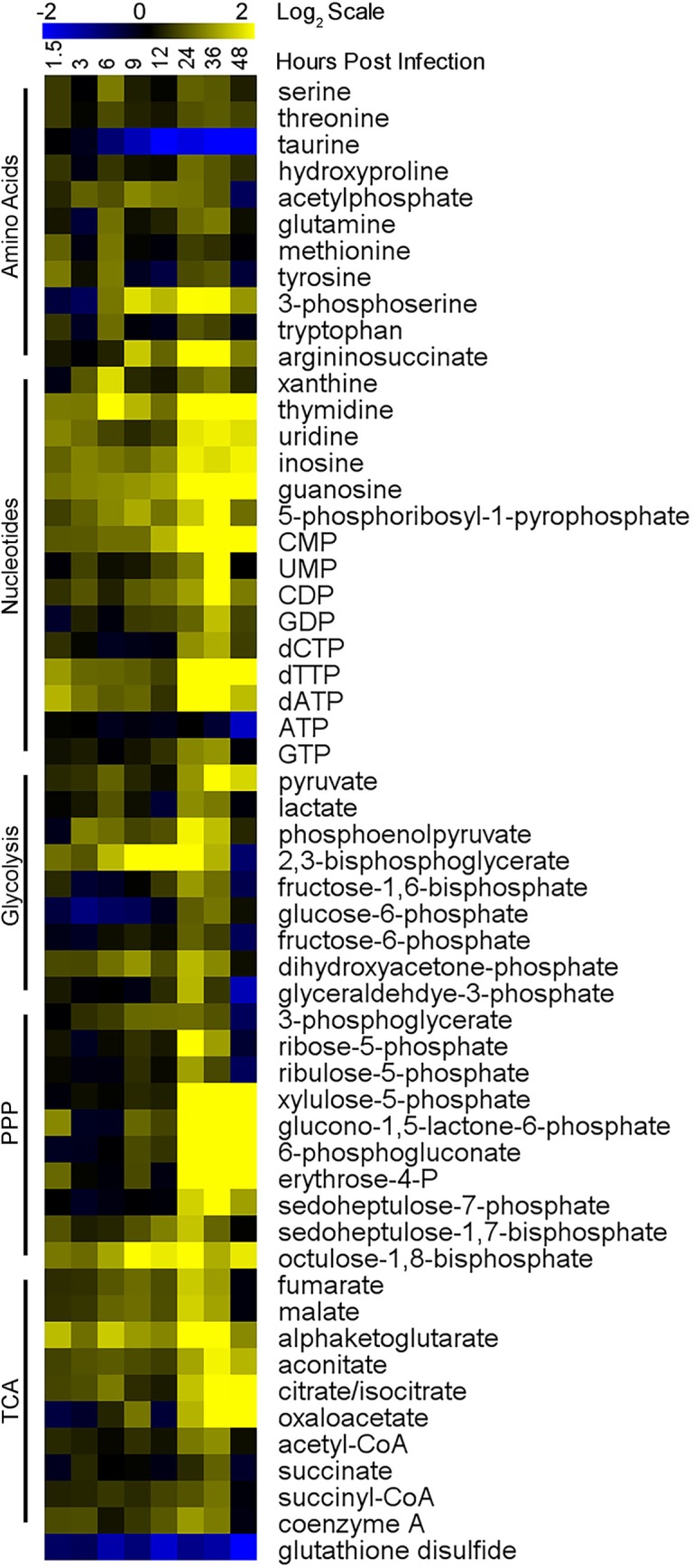
*T*. *gondii* infection changes the metabolome. Heatmap shows metabolite abundance over 48 hours of *T*. *gondii* infection. Triplicate infected and uninfected dishes of HFFs were metabolically quenched and metabolites were extracted at 8 times points (1.5, 3, 6, 9, 12, 24, 36, and 48 HPI). Metabolomes were quantified using HPLC-MS and metabolites were identified with known standards. Infected sample abundances were averaged and normalized to the average uninfected abundance then log base 2 transformed (Log_2_(Infected Abundance/Uninfected Abundance)) with blue being less abundant and yellow more abundant. Values represented in this heat map are listed in S3 Table.

We conducted a temporal mass spectrometry-based analysis of metabolism during *T*. *gondii* infection (MOI 0.625) over 48 hours using a fibroblast tissue culture infection model. Metabolome experiments were conducted with infected and uninfected samples at each time point for three independent experiments. Metabolism was rapidly quenched and metabolites were extracted from infected and uninfected dishes at eight time points (1.5, 3, 6, 9, 12, 24, 36, and 48 hours post infection). The metabolome in each sample was quantified using Ultra High-Pressure Liquid Chromatography paired with Mass Spectrometry (HPLC-MS). Metabolites were identified through comparison to the mass and retention time of standards run on the same HPLC-MS [22,23]. The first two independent experiments were performed with single plates per time point (S1 Fig) and the third experiment was done with samples in triplicate to allow for statistical analysis (S1 Table). Many metabolites had significant variations in abundance between infected and uninfected samples (Fig 1). Metabolite abundance was altered for intermediates of central metabolic pathways including glycolysis, the TCA cycle, the PPP, amino acid and nucleotide synthesis.

**Fig 2:**
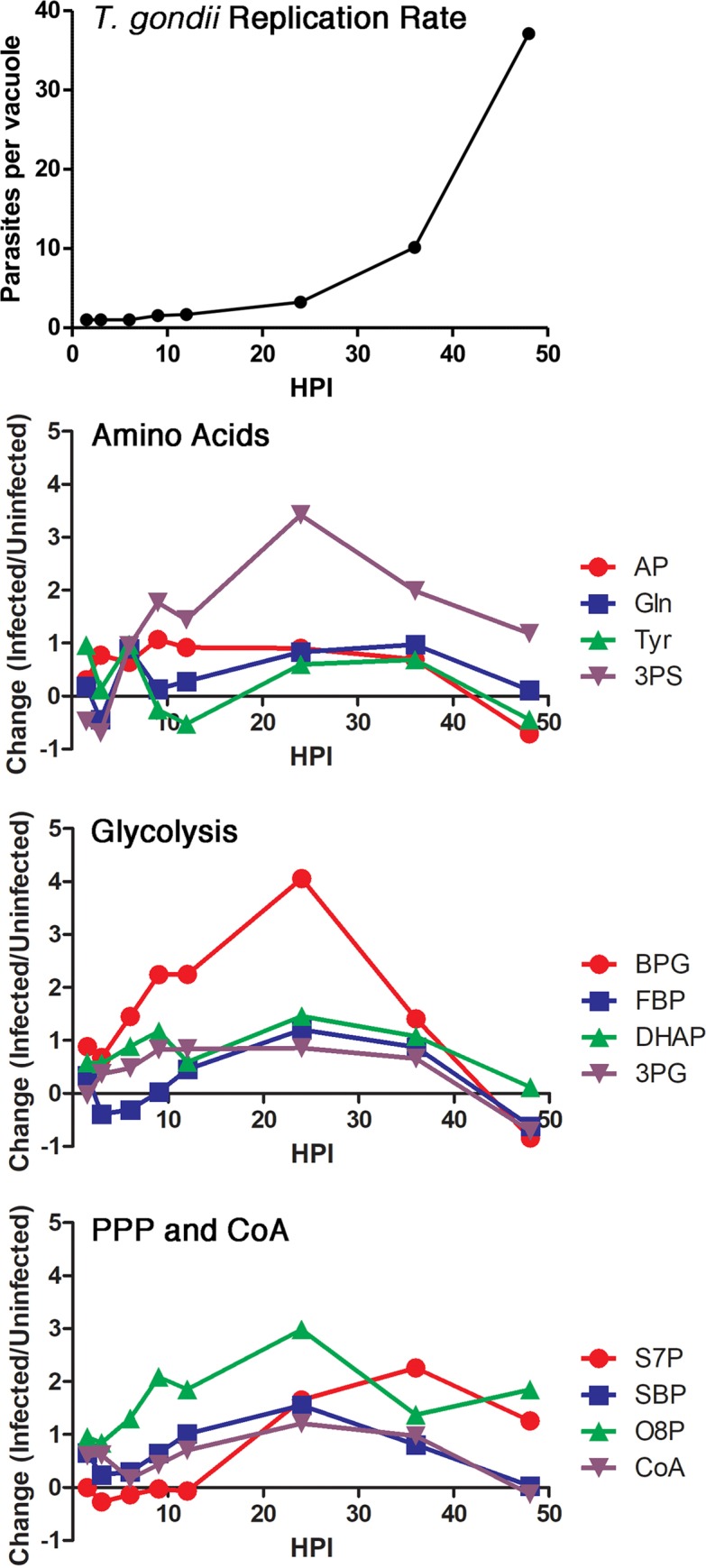
Metabolites that do not follow the *T*. *gondii* replication rate. In the top panel, *T*. *gondii* replication rate was measured during the metabolomics time course. Fibroblasts grown on coverslips were infected under the same conditions as the metabolomics time course. At each time point, infected cells were fixed, permeabilized and mounted with DAPI. *T*. *gondii* nuclei were counted in 100 cells for each time point and averaged. Error bars represent a 95% confidence interval. In the lower three panels, the 12 metabolites whose abundances do not follow the *T*. *gondii* replication rate are shown. The y-axis is Log_2_ fold change infected/uninfected. The second panel shows metabolites involved in amino acid synthesis, acetylphosphate (AP, red circles), glutamine (Gln, blue squares), tyrosine (Tyr, green triangles) and 3-phosphoserine (3PS, purple triangles). The third panel shows metabolites involved in glycolysis including 2,3-bisphosphoglycerate (BPG, red circles), fructose-1,6-bisphosphate (FBP, blue squares), dihydroxyacetone phosphate (DHAP, green triangles) and 3-phosphoglycerate (3PG, purple triangles). The last panel shows metabolites involved in the PPP, sedoheptulose-7-phosphate (S7P, red circles), sedoheptulose-1,7-bisphosphate SBP, blue squares) and octulose-8-phosphate (O8P, green triangles). Also shown is Coenzyme A (CoA, purple triangles).

To analyze whether these metabolite changes were directly linked to increases in parasite numbers, we measured the parasite replication rate in parallel under identical conditions at the same time points. A normal progression of replication for type II ME49 was observed, with the largest increases in parasites between 36 to 48 hours post infection (Fig 2). We then calculated the abundance for each metabolite per parasite, and while most metabolites increased proportional to the number parasites, 12 metabolites peaked in abundance prior to the onset of exponential parasite growth (Fig 2). These metabolites are by synthesized in several different metabolic pathways, highlighting that multiple pathways are upregulated before parasite replication.

### Taurine release

The only two metabolites which decreased in abundance consistently over infection were taurine and glutathione disulfide. While glutathione disulfide depletion was likely due to high levels of oxidative metabolism, we initially believed that taurine depletion may have been due to a *T*. *gondii* taurine degradation pathway. To test for taurine degradation a metabolomics time course was repeated using media supplemented with 2mM ^15^N-Taurine. Several bacterial species are known to degrade taurine via a pathway that results in the nitrogen being incorporated into alanine and other amino acids [24,25]. However, no ^15^N was detected in any intracellular metabolite except taurine, and taurine levels did not decrease as previously observed. This result did not rule out taurine conjugation by *T*. *gondii*, or degradation via another route, but it did indicate that the parasite was not likely using taurine to fuel amino acid synthesis.

These findings led us to believe that taurine depletion may be due to secretion instead of degradation. To test for taurine secretion media samples were taken from infected dishes with either 0 taurine metabolomic media or media supplemented with a physiological level (44 μM) of taurine [26]. Intracellular and extracellular samples were taken over the usual time course and analyzed using our standard metabolomics methodology. We discovered that in media with no added taurine the only metabolite with an altered abundance was taurine, which increased significantly in abundance in a pattern that mimicked the drop in intracellular taurine (S2 Fig). In media supplemented with 44μm taurine, increases in taurine in the media could only be detected at 48 hours post infection. Thus, the changes in taurine levels are likely due to secretion of taurine into the media, and not due to parasite metabolism. The taurine release is probably associated with *T*. *gondii* infection increasing phospholipase A_2_ activity [27,28] as PLA_2_ activity causes taurine secretion [29].

### Changes in amino acid metabolism

Several amino acids and their biosynthetic intermediates had moderately increased abundance at 24 and 36 hours post infection (HPI) (Fig 1). *T*. *gondii* is auxotrophic for tryptophan and arginine [10,11], so these amino acids must be scavenged from the host cell. Tryptophan abundance increased over the infection time course. Likewise, argininosuccinate, the penultimate metabolite in arginine synthesis, increased in abundance in infected cells. The infected host transcriptome compared to the uninfected control indicates possible explanations for these metabolomic shifts. Humans cannot synthesize tryptophan and LAT1 is the transporter responsible for tryptophan uptake. Transcription of LAT1 increases during infection, particularly from 6 to 48 HPI when expression is 5-fold higher than the uninfected control (Table 1). Arginino Succinate Synthase 1 (ASS1), which catalyzes the rate limiting step in arginine synthesis, was highly expressed throughout infection, including a period from 9 to 24 HPI when expression was approximately double that of the uninfected cells (Table 1). The cause of increased ASS1 expression is unknown but ASS1 activity is critical to *T*. *gondii* replication as previous studies have shown arginine synthesis is essential to lytic parasite growth [10].

**Table 1:** Host mRNA abundance of genes of interest (fold change infected/uninfected). mRNA fold change of host genes of interest in was calculated for infected cells versus uninfected cells. RNA samples were isolated from infected dishes paired to infected metabolomics dishes and from an uninfected control dish. RNAseq was performed to generate the transcriptome and fold change (infected/uninfected) determined. Infection increased expression of multiple host metabolic genes in central pathways including glycolysis, the PPP, nucleotide metabolism, and amino acid synthesis and transport. Large amino acid transporter 1 (LAT1), argininosuccinate synthase 1 (ASS1), phosphoglycerate dehydrogenase (PHGDH), methylenetetrahydrofolate dehydrogenase (MTHFD1), 5-phosphoribosyl-1-pyrophosphate synthase (PRS1), hexose kinase (HK), pyruvate kinase (PK), fructose-1,6-bisphosphate aldolase 1 (FBA1), lactate dehydrogenase A (LDHA), bisphosphoglycerate mutase (BPGM), aconitase (ACN), isocitrate dehydrogenase (IDH), alpha-ketoglutarate dehydrogenase (A-KGDH), succinate reductase (SCR), fumarase (FUM), malate dehydrogenase (MDH), citrate synthase (CS), succinyl-CoA synthetase (SCS), glucose-6-phosphate dehydrogenase (G6PD), 6-phosphogluconolactonase (6PGL), 6-phosphogluconate dehydrogenase (6PGD), ribose-5-phosphate isomerase (R5PI), ribulose-5-phosphate epimerase (Ru5PE), transaldolase (TA), transketolase (TK).

Gene	1.5	6	9	12	24	36	48
LAT1	5.49	11.5	5.89	11.8	6.31	4.89	51.7
ASS1	1.27	1.92	1.96	2.39	2.29	1.48	0.35
PHGDH	0.80	2.30	1.14	3.55	5.46	4.90	1.40
MTHFD1	0.77	1.47	1.30	1.59	2.06	2.58	2.05
PRS1	2.27	2.26	2.18	2.21	3.12	3.94	2.64
MYC	1.99	6.07	4.85	5.23	3.04	1.76	4.30
HK	1.36	3.20	2.85	2.65	1.50	1.81	2.88
PK	1.25	1.44	1.30	1.44	1.33	1.51	1.12
FBA1	1.52	2.31	2.23	2.10	2.47	2.81	3.17
LDHA	1.79	2.85	2.53	2.22	2.34	2.51	0.91
BPGM	1.50	1.36	1.56	0.71	0.52	0.44	0.18
CAN	1.08	0.92	0.95	0.89	0.58	0.66	0.56
IDH	1.05	0.97	1.12	1.03	1.03	1.18	0.39
A-KGDH	0.79	0.96	0.99	0.83	0.64	0.50	1.48
SCR	0.99	0.68	0.78	0.79	0.90	1.19	0.22
FUM	0.69	0.62	0.63	0.57	0.65	0.59	0.20
MDH	0.93	0.91	0.93	0.94	1.02	0.98	0.34
CS	1.01	2.65	1.50	2.18	1.28	1.26	4.44
SCS	0.70	0.49	0.42	0.57	0.56	0.31	0.48
G6PD	1.08	1.35	1.73	1.63	1.28	1.15	0.42
6PGL	0.78	0.66	0.62	0.75	0.95	0.83	0.18
6PGD	0.86	1.55	1.33	1.68	2.31	3.25	1.55
R5PI	1.29	2.60	2.54	2.88	2.50	2.05	1.79
Ru5PE	0.58	0.95	0.57	1.13	0.76	1.11	1.10
TA	1.03	1.32	1.42	1.62	2.46	2.19	0.35
TK	0.59	0.65	0.61	0.52	0.59	0.68	0.32

### Nucleotide synthesis increases

A wide range of metabolites involved in nucleotide synthesis dramatically increased abundance in infected cells, with most changes occurring in the 24–48 HPI range (Fig 1). These increased metabolites include guanosine, inosine, uridine, deoxynucleoside thymidine, xanthine and 5-phosphoribosyl-1-pyrophosphate (PRPP), which is an essential metabolite in purine and pyrimidine synthesis and salvage. Additionally, CMP, dTTP and dATP were significantly upregulated at most infection time points, while UMP, CDP, GDP, GTP and dCTP had significantly increased abundance only 1 or 2 infection time points. In infected cells, the important purine synthetic enzyme tetrahydrofolate synthase (MTHFD1) was upregulated compared to uninfected cells from 6 to 48 HPI (Table 1). PRPP Synthase (PRS1), which is essential for purine and pyrimidine synthesis, was upregulated in infected host cells throughout infection, with a peak of 4 fold increased expression at 36 HPI (Table 1). Transcription of the regulatory protein Myc, which activates nucleotide synthesis, was increased at every time point in infected cells, reaching 6 fold increased transcription over uninfected cells at 6 HPI (Table 1). This result was expected given previous work demonstrating *T*. *gondii* infection induces increased Myc expression and activity [30]. Two parasite enzymes which are essential to nucleotide salvage, hypoxanthine-guanine phosphoribosyl transferase and adenosine kinase, were consistently expressed throughout infection, as was *T*. *gondii* PRS1 which creates the PRPP needed for nucleotide synthesis and salvage (Table 2).

**Table 2:** *T*. *gondii* mRNA abundance of genes of interest (Unprocessed FPKM values). mRNA abundance of *T*. *gondii* genes of interest in raw FPKM values. RNA samples were isolated from infected dishes paired to infected metabolomics dishes and RNAseq was performed to generate the transcriptomes. Genes throughout central metabolism are expressed at a high level throughout the parasite lytic cycle. Phosphoglycerate dehydrogenase (PHGDH), hypoxanthine phosphoribosyltransferase (HXGPRT), adenosine kinase (AK), 5-phosphoribosyl-1-pyrophosphate synthase (PRS1), hexose kinase (HK), pyruvate kinase (PK), fructose-1,6-bisphosphate aldolase (FBA), lactate dehydrogenase 1 (LDH1), aconitase (ACN), alpha-ketoglutarate dehydrogenase (A-KGDH), succinate reductase (SCR), fumarase (FUM), isocitrate dehydrogenase 2 (IDH—TCA), isocitrate dehydrogenase 1 (IDH-API), malate dehydrogenase (MDH), citrate synthase (CS), succinyl-CoA synthetase (SCS), glucose-6-phosphate dehydrogenase putative 1 (G6PD 1), glucose-6-phosphate dehydrogenase putative 2 (G6PD 2), 6-phosphogluconate dehydrogenase putative 1 (6PGD 1), 6-phosphogluconate dehydrogenase putative 2 (6PGD 2), ribose-5-phosphate isomerase (R5PI), ribulose-5-phosphate epimerase (Ru5PE), transaldolase (TA), transketolase (TK), sedoheptulose bisphosphatase (SBPASE).

Gene	TGME49_	1.5	6	9	12	24	36	48
PHGDH	239820	131	221	228	217	215	190	157
HXGPRT	200320	7.85	23.3	21.2	17.7	20.9	8.72	56.6
AK	250880	21.3	52.9	55.1	48.3	51.0	40.9	16.9
PRS1	220100	28.2	38.5	34.2	28.8	23.5	16.6	35.9
HK	265450	42.4	72.7	79.9	68.5	70.3	60.7	59.8
PK	256760	50.0	116	95.7	96.8	93.3	79.3	116
FBA	236040	576	580	593	585	670	671	188
LDH1	232350	351	361	394	337	349	310	614
ACN	226730	27.9	33.0	24.1	37.0	26.9	21.5	52.8
A-KGDH	244200	18.3	20.9	18.5	25.2	19.9	14.8	32.4
SCR	215280	27.8	38.6	28.2	37.7	28.9	21.6	39.5
FUM	267330	8.63	15.7	18.4	16.2	13.8	9.28	13.0
IDH-TCA	266760	56.1	110	115	112	106	94.0	42.3
IDH-API	313140	3.38	44.2	23.9	45.0	43.7	38.9	13.3
MDH	318430	79.4	259	248	280	309	218	52.7
CS	203110	24.1	39.0	39.5	44.6	42.4	38.2	26.8
SCS	290600	75.8	103	114	116	119	93.5	61.0
G6PD 1	278830	39.5	41.9	27.9	40.1	21.1	21.3	127
G6PD 2	294200	710	435	434	364	292	363	466
6PGD 1	242600	0.00	0.00	0.00	0.08	0.00	0.17	0.09
6PGD 2	307850	4.69	22.3	34.6	19.5	25.2	23.3	13.9
R5PI	239310	16.8	31.1	31.0	35.4	23.5	22.6	16.9
Ru5PE	247670	10.0	14.4	21.4	12.3	15.2	14.7	15.6
TA	229360	77.6	122	134	103	112	126	47.6
TK	318310	6.96	40.3	38.1	39.0	35.8	22.0	27.6
SBPASE	235700	57.1	98.8	118	91.3	105	112	38.5

### Glycolysis increases

Glycolytic intermediates were generally more abundant in the infection metabolome, apart from 48 HPI when many were depleted. This result is expected given previous work demonstrating a highly active *T*. *gondii* glycolytic pathway and the energetic burden of infection on host cells [2,4,7,31]. Host hexose kinase (HK) and pyruvate kinase (PK) were transcriptionally upregulated in infected cells at every time point during infection (Table 1) and were highly transcribed throughout infection in *T*. *gondii* with FPKM values regularly at or above 50 (Table 2). Dihydroxyacetone phosphate (DHAP) and lactate were also increased in abundance in the infection metabolome. DHAP is a product of fructose 1,6-bisphosphate and lactate is synthesized by lactate dehydrogenase. Both fructose 1,6-bisphosphate and lactate dehydrogenase were transcriptionally upregulated in infected host cells and highly transcribed throughout infection in *T*. *gondii*, highlighting that both host and parasite likely contributed to the increases in DHAP and lactate (Tables 1 and 2). The increase in 2,3-bisphosphoglycerate (2,3-BPG) was intriguing because it’s synthesis in red blood cells by the rapoport-luebering shunt decreases ATP production from glycolysis by 50% [32]. 2,3-BPG is well-known as an allosteric regulator of oxygen avidity to hemoglobin, but 2,3-BPG is found in other tissues and may play a role in the control of protein degradation rates [33]. In infected HFF cells, bisphosphoglycerate mutase (BPGM), the main source of 2,3-BPG, is transcriptionally upregulated during the first twelve hours of infection (Table 1). *T*. *gondii* BPGM has yet to be identified, which makes it difficult to estimate the parasite contribution to 2,3-BPG synthesis. Future studies of 2,3-BPG will determine if it is produced by the host or parasites and why this seemingly wasteful product is synthesized.

### Changes in the TCA cycle

**Fig 3:**
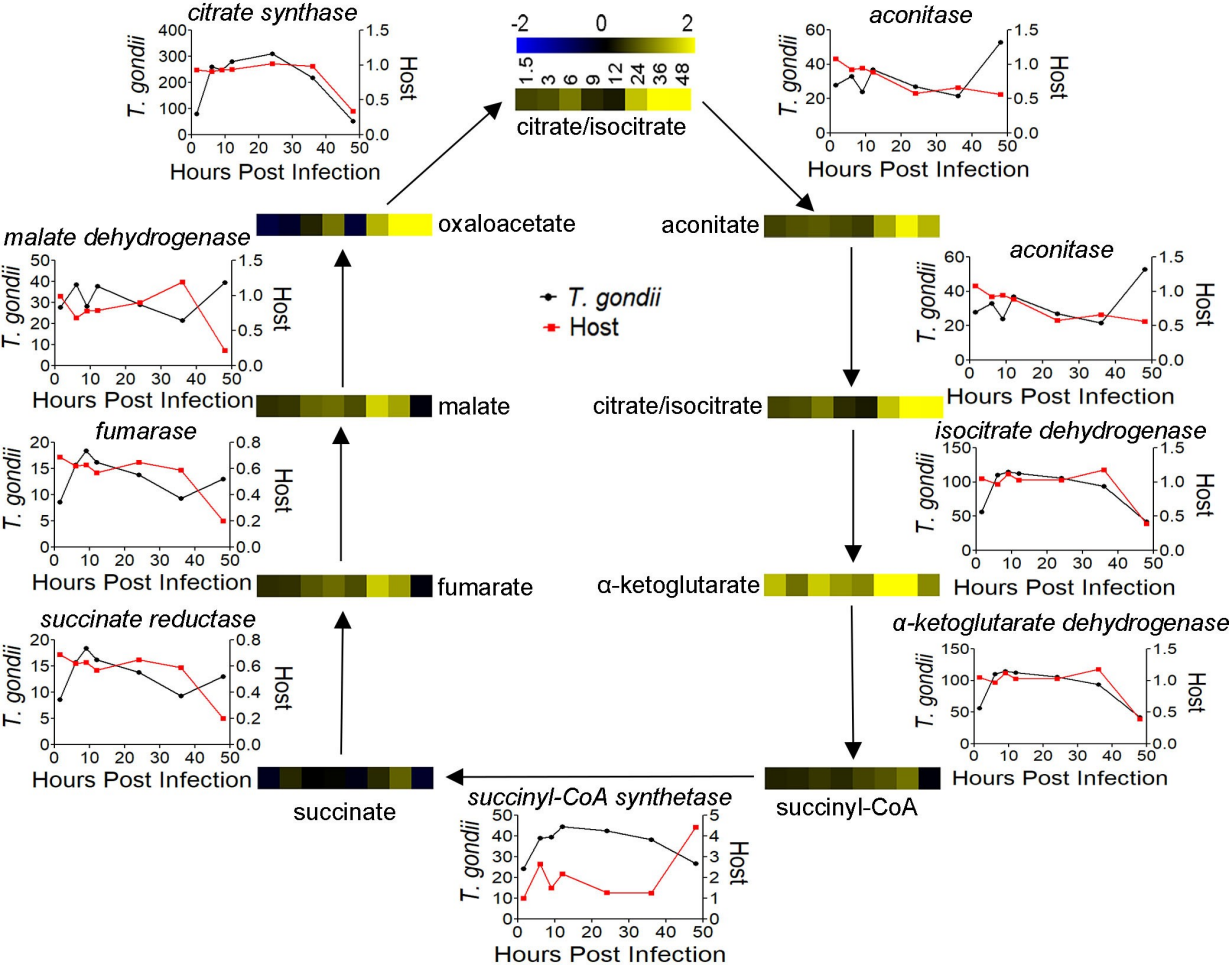
*T*. *gondii* infection TCA cycle metabolome and transcriptome. Heatmaps show the abundance of each TCA cycle intermediate over 48 hours with blue being less abundant and yellow more abundant. The line graphs represent mRNA abundance for the host (red) and *T*. *gondii* (black) TCA cycle enzyme (names italicized). Host expression is shown as fold change (infected/uninfected) and *T*. *gondii* expression is shown in FPKM values.

*T*. *gondii* infection of fibroblasts increases the abundance of multiple TCA cycle intermediates, including citrate/isocitrate, oxaloacetate, aconitate and α-ketoglutarate (Fig 3). Changes in abundance range from 2–8 fold increases over uninfected cells from 24 to 48 HPI. Malate, fumarate, succinyl-CoA and succinate were also increased in abundance 1 to 2 fold over uninfected cells from 24 to 48 HPI. *T*. *gondii* uses an γ-aminobutyric acid (GABA) shunt to fuel the TCA cycle [2], but changes in GABA abundance over the time course of infection were not statistically significant. Two key metabolites, acetyl-CoA and Coenzyme A, which are essential to fueling the TCA cycle, are similarly more abundant during late *T*. *gondii* infection.

Our *T*. *gondii* transcriptome supports previous findings, with all enzymes in the TCA cycle expressed throughout infection (Table 2). Critically, the enzyme that catalyzes the rate limiting step of the TCA cycle, Isocitrate dehydrogenase, is highly expressed at approximately 100 FPKM from 6 to 36 HPI. The enzyme which is responsible for generating acetyl-CoA to fuel the TCA cycle, branched-chain α-ketoacid dehydrogenase (BCKDH), is also expressed at a high level throughout infection [34]. There are secondary forms of two TCA cycle enzymes, aconitase and isocitrate dehydrogenase, which are localized to the apicoplast where they produce reducing equivalents for biosynthetic pathways [34,35]. While these enzymes are not involved in the mitochondrial TCA cycle, they share intermediates and contribute to the observed TCA cycle phenotype. The apicoplast-aconitase originates from the same gene as the mitochondrial aconitase but is localized to both organelles through an unusual signal sequence [34,36]. The apicoplast isocitrate dehydrogenase is highly expressed from 6 to 48 HPI and likely plays a role in the increase in abundance of citrate/isocitrate and α-ketoglutarate.

While the *T*. *gondii* TCA cycle is known to be active during infection, the host cycle has not been as heavily studied. Transcription of most host TCA cycle genes does not increase during infection, except to be decreased at 48 hours post infection, likely due to some early lysis (Table 1). Host succinate dehydrogenase, succinyl-CoA synthetase, and fumarase are less transcribed in infected cells throughout, while pyruvate dehydrogenase, α-ketoglutarate dehydrogenase, aconitase, malate dehydrogenase, citrate synthase and isocitrate dehydrogenase are at or below the expression of uninfected control cells.

### The Pentose Phosphate Pathway (PPP) is metabolically altered and transcriptionally activated in *T*. *gondii* and the host

**Fig 4:**
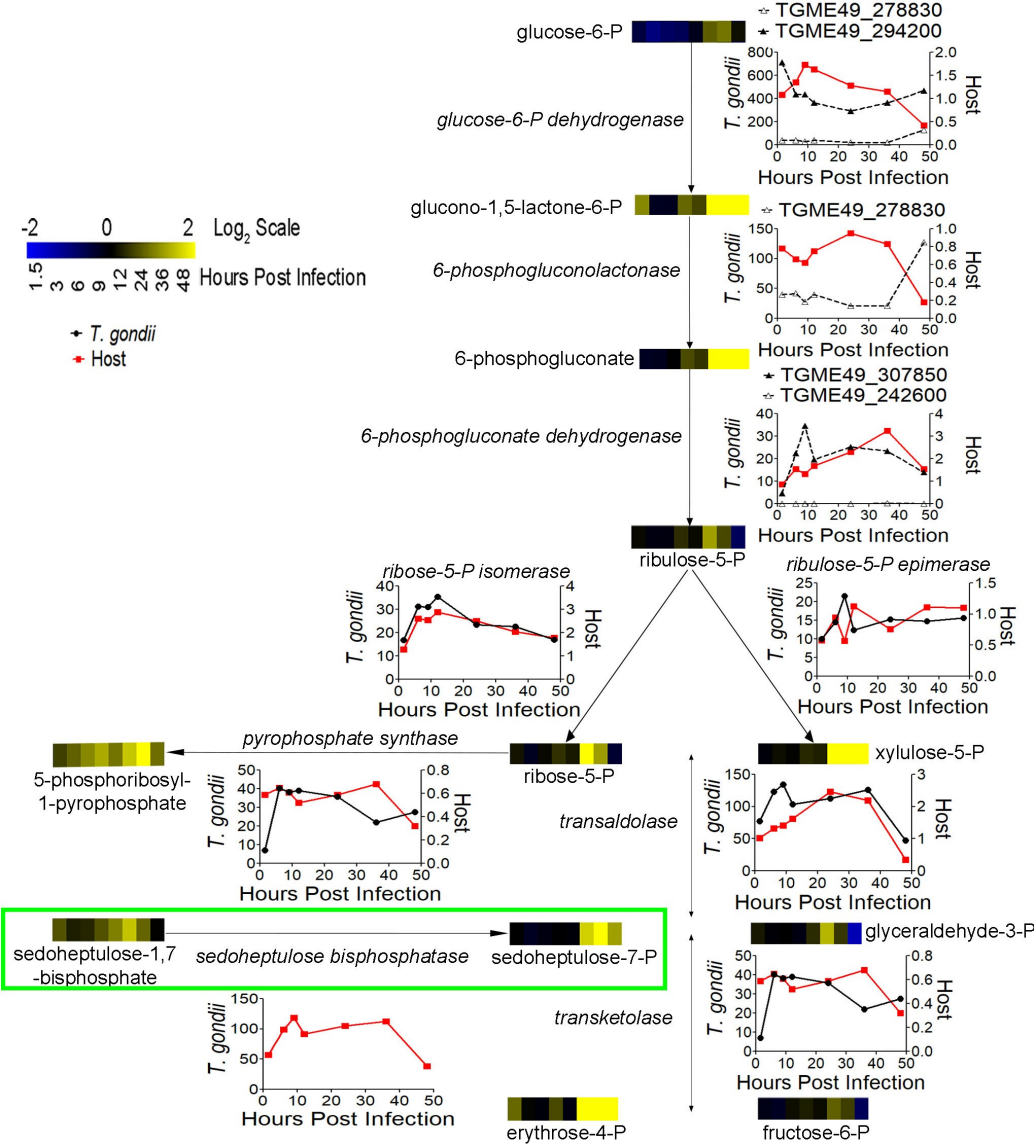
*T*. *gondii* infection PPP metabolome and transcriptome. Heatmaps show the abundance of each PPP intermediate over 48 hours. The graphs represent mRNA abundance for host (red) and *T*. *gondii* (black) PPP cycle enzyme (names italicized). Host expression is shown as fold change (infected/uninfected) and *T*. *gondii* expression is in FPKM values. Putative *T*. *gondii* genes in the oxidative PPP are noted with dashed lines. The *T*. *gondii* specific SBPase activity has been added to the PPP and boxed in bright green.

Multiple intermediates in the oxidative and non-oxidative PPP had increased abundance in the infection metabolome. Glucono-1,5-lactone-6-phosphate and gluconate-6-phosphate in the oxidative pathway are increased in abundance from 2.5 to 4.9 fold over uninfected cells from 24 to 48 HPI (Fig 4). A similar pattern was found in xylulose-5-phosphate abundance, which was 2.8 to 3.9 fold more abundant from 24 to 48 HPI. Sedoheptulose-7-phosphate (S7P) was 1.6 to 2.2 fold more abundant from 24 to 48 HPI, with similar, but slightly earlier changes occurring in sedoheptulose-1,7-bisphosphate (SBP) (Fig 1). This is the first report documenting the presence of SBP in *T*. *gondii* infected cells, although we cannot determine if the parasite or host is synthesizing this metabolite. While standards are not commercially available for octulose-8-phosphate (O8P) and octulose-1,8-bisphosphate (OBP), peaks matching the exact molecular masses of O8P and OBP displayed similar patterns to S7P and SBP. The earlier increase in SBP levels compared to S7P is suggestive of phosphatase activity. Glyceraldehyde-3-phosphate was moderately more abundant in infected samples but to a much lesser degree than other PPP intermediates and shifts in abundance were not limited to late infection. Given the dual role of glyceraldehyde-3-phosphate as an intermediate in glycolysis and the PPP, it is understandable that shifts in its abundance do not match up perfectly with other metabolites in either pathway.

Host enzymes of the oxidative and non-oxidative PPP were generally expressed throughout infection, except at 48 hours post infection, many decreased likely due to early lysis (Table 1). The rate limiting enzyme of the oxidative pathway, glucose-6-phosphate dehydrogenase, is expressed at or slightly above uninfected levels for the majority of infection, 6 to 36 HPI. Messenger RNA abundance of 6-phosphogluconolactone dehydrogenase, which catalyzes the third reaction in the oxidative PPP, is higher in infected cells from 6 to 48 HPI, peaking at 3.3 fold at 36 HPI. Both steps are responsible for generating NADPH and their expression suggests an activate host oxidative PPP. The two enzymes of the non-oxidative PPP, transketolase and transaldolase, have minor regulation but are expressed throughout infection.

Transcriptomic analysis of the *T*. *gondii* PPP is complicated because the genes of the oxidative PPP are uncharacterized. Based on the current genome annotation, there are two putative glucose-6-phosphate dehydrogenases (TGME49_278830 and TGME49_294200) and two putative 6-phosphogluconate dehydrogenases (TGME49_242600 and TGME49_307850). One of the putative glucose-6-phosphate dehydrogenase genes also has putative phosphogluconolactonase activity. This activity would agree with recent findings that *Plasmodium* possesses a bifunctional enzyme responsible for the first two steps of the oxidative PPP [37]. The putative bifunctional glucose-6-phosphate dehydrogenase-phosphogluconolactonase was expressed throughout infection, with FPKM values in the 20 to 40 range, while the glucose-6-phosphate dehydrogenase without putative secondary activity was highly expressed with FPKM’s over 300 for most of infection (Table 2). In contrast, only one of the 6-phosphogluconate dehydrogenases was expressed throughout infection, with FPKMs in the 20–30 range, while the other had no expression at multiple time points. Given that at least one of each of the putative oxidative PPP genes are expressed throughout infection we believe that the *T*. *gondii* oxidative PPP is active during infection. In the non-oxidative PPP *T*. *gondii* transketolase is expressed at 20 to 40 FPKM from 6 to 48 HPI while transaldolase is highly expressed throughout infection with FPKM values over 100 from 6 to 36 HPI.

### *T*. *gondii* genome contains a predicted Sedoheptulose-1,7-bisphosphatase

As described earlier, the increased abundance of SBP and OBP before than S7P and O8P, lead us to investigate whether these metabolites were synthesized sequentially. Sedoheptulose-1,7-bisphosphatase (SBPase) in *Saccharomyces cerevisiae* is known to generate S7P and O8P from SBP and OBP, respectively [19]. SBPase activity has so far not been found in warm-blooded animals [19–21]; however, *T*. *gondii* has a putative SBPase (TGME49_235700) and genetic analysis has identified a likely SBPase in the protozoan parasite *Trypanosoma brucei* [38].

We generated an amino acid sequence alignment using the predicted amino acid sequence of TGME49_235700 with the *T*. *brucei* SBPase as well as the two known SBPase enzymes from the fungal and plant model organisms *S*. *cerevisiae* and *Arabidopsis thaliana* (S3 Fig). The *T*. *gondii*, *T*. *brucei* and *A*. *thaliana* SBPases have a higher level of sequence similarity to each other than they have with the *S*. *cerevisiae* enzyme. The only major difference between *T*. *gondii*, *T*. *brucei* and *A*. *thaliana* SBPases is the 74 amino acid chloroplast transit peptide on the *A*. *thaliana* SBPase. The residues for metal and substrate binding have been predicted on the on the *A*. *thaliana* SBPase and are all conserved with the *T*. *gondii* and *T*. *brucei* SBPases (S3 Fig). These similarities lead us to hypothesize that *T*. *gondii* SBPase, as predicted for the *T*. *brucei* enzyme, is of plant origin [38]. *A*. *thaliana* SBPase is involved in Calvin cycle reactions [39]; however, this pathway is not present in *T*. *gondii*, suggesting possible new roles for this enzyme.

### [6-^13^C_1_]glucose labeling indicates that *T*. *gondii* possesses Sedoheptulose Bisphosphatase activity

SBPase activity can be assayed for using [6-^13^C_1_]glucose labeling. In the absence of SBPase, S7P can only be present in the unlabeled (M+0, containing only ^12^C atoms) or M+1 (containing one ^13^C atom) labeled forms, while cells with SBPase activity will have M+2 (containing two ^13^C atoms) labeled forms labeled forms [19, simplified carbon flow diagram S4 Fig]. Fructose bisphosphate aldolase synthesis of sedoheptulose-1,7-bisphosphate produces M+2 SBP, which results in M+2 S7P if SBPase is present to dephosphorylate SBP. Under normal cellular conditions with high glycolytic flux, SBPase is the only reaction that results in double labeling (M+2) of S7P, when cells are grown in the presence of [6-^13^C_1_]glucose. One difficulty in adapting this technique to *T*. *gondii* is the presence of an active gluconeogenic futile cycle between fructose-1,6-bisphosphate and fructose-6-phosphate [40]. This reaction would result in some double labeling of sedoheptulose-7-phosphate proportionate to the level of double labelled F6P.

**Fig 5:**
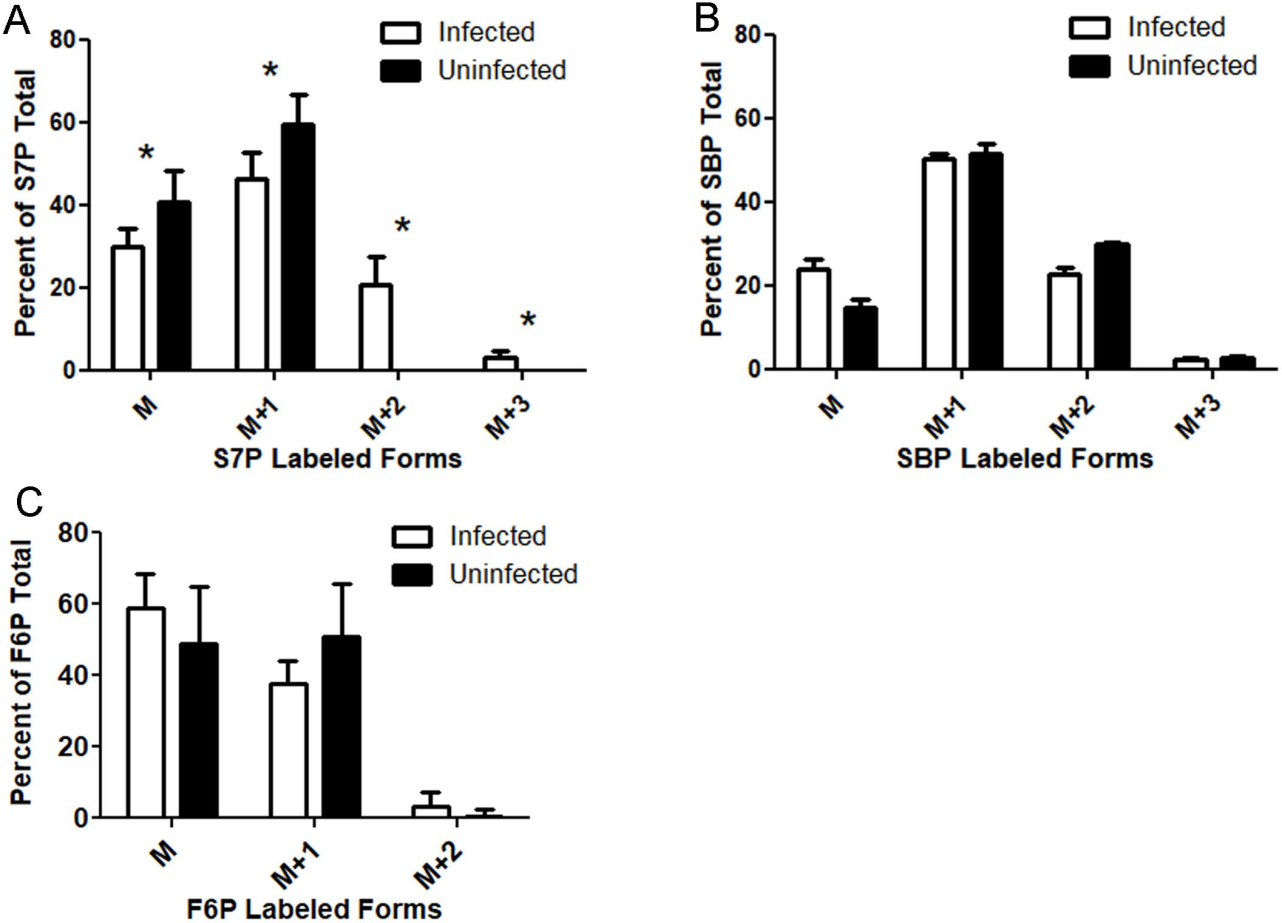
[6-^13^C_1_]glucose labeling indicates *T*. *gondii* possesses SBPase activity. (A) Mass (M) of S7P as a percentage of the total in infected (white bars) and uninfected (black bars) cells. M+1 is S7P containing one ^13^C, M+2 is S7P containing two ^13^C and M+3 is S7P containing three ^13^C. Asterisks mark significant differences (p<0.05, two tailed t-test) between infected and uninfected samples. Error bars are a 95% confidence interval. Total S7P ion counts in *T*. *gondii* infected cells were 106700 ± 13570 and in uninfected the S7P ion counts were 42154 ± 3659, n = 3. (B) Mass (M) of SBP as a percentage of the total in infected (white bars) and uninfected (black bars) cells. M+1 is SBP containing one ^13^C, M+2 is SBP containing two ^13^C, and M+3 is SBP containing three ^13^C. No significant differences were seen between infected and uninfected. Error bars are a 95% confidence interval. Total SBP ion counts in *T*. *gondii* infected cells were 2729400 ± 406700 and in uninfected the SBP ion counts were 1094000 ± 211500, n = 3. (C) Mass (M) of F6P as a percentage of the total in infected (white bars) and uninfected (black bars) cells. M+1 is F6P containing one ^13^C, M+2 contains two ^13^C, and M+3 contains three ^13^C. No significant differences were seen between infected and uninfected. Error bars are a 95% confidence interval.

Triplicate infected and uninfected dishes were grown for 24 hours with [6-^13^C_1_]glucose media. Uninfected S7P was 48% unlabeled and 52% M+1 labeled, exhibiting no SBPase activity (Fig 5A). Infected S7P was 40% unlabeled, 46% M+1 labeled, and 14% M+2 labeled, suggesting SBPase activity (Fig 5A). The absence of +2 labeled S7P in the uninfected cells was not due to a lack of M+2 labeled SBP (Fig 5B). Uninfected cells had 30% M+2 labeled, and 2.8% M+3 labeled SBP, demonstrating that uninfected cells do not convert SBP into S7P (Fig 5B). While it is difficult to compare the percent of labelling between F6P and S7P because it is not clear how much of each metabolite is from host and how much is from parasite, F6P was found to be 3.4% +2 labeled in infected cells. This small amount of M+2 FBP is unlikely to result in the 14% of +2 labeled S7P, which indicates that *T*. *gondii* likely possesses a second source of S7P synthesis via SBPase (Fig 5C).

### Expression of *T*. *gondii* SBPase in HeLa cells shows SBPase activity

**Fig 6:**
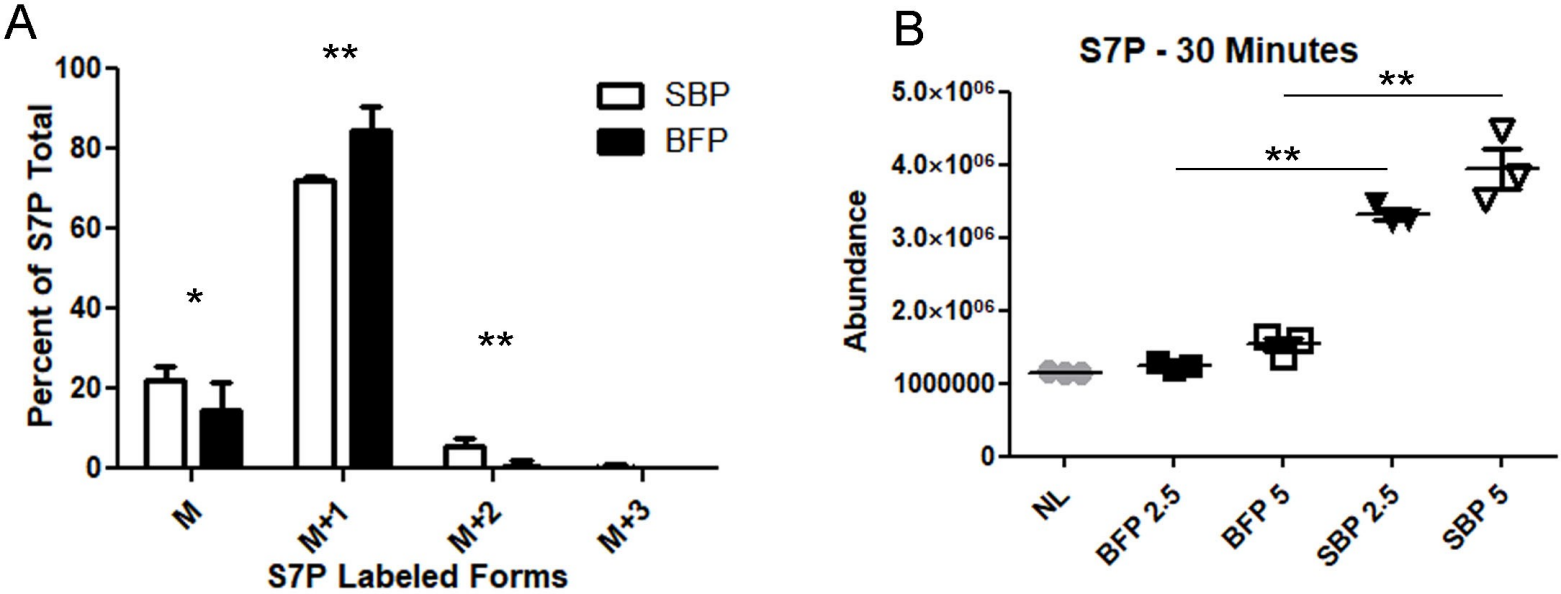
Expression of *T*. *gondii* SBPase in HeLa results in SBPase activity. (A) Average S7P isotope composition for HeLa cells expressing SBPase (SBPase, white bars) or BFP (black bars) as a percentage (M = parental unlabeled, M+1 = mono ^13^C labeled, M+2 = double ^13^C labeled). Asterisks mark significant differences in abundance (* p<0.05, ** p<0.01 two tailed t-test). Error bars are a 95% confidence interval. Total S7P ion counts in SBPase expressing HeLa cells were 289000 ± 111300 and in expressing HeLa cells the BFP total S7P ion counts were 241650 ± 97360. Total SBP ion counts in SBPase expressing HeLa cells were only 461 ± 368, but in expressing HeLa cells the BFP total SBP ion counts were 254077 ± 117590. Two independent experiments in triplicate were performed. (B) SBP was synthesized from erythrose-4-phosphate, dihydroxyacetone phosphate, and fructose bisphosphate aldolase for 15 minutes. S7P abundance was measured after incubation with either no lysate (grey circles), BFP expressing HeLa cell lysate (black shapes), or SBPase expressing HeLa cell lysate (white shapes). Incubation was carried out with either 2.5 μg (squares) or 5 μg (triangles) lysate for 30 minutes. Asterisks mark significant differences in abundance (**p<0.01 two tailed t-test). Error bars mark mean and a 95% confidence interval. Two independent experiments in triplicate were performed (replicate S6 Fig).

To examine the activity of the putative *T*. *gondii* SBPase (TGME49_235700), a lentiviral expression system was used to stably incorporate the gene into the genome of HeLa cells. As a control, a separate population of HeLa cells were transduced with Blue Fluorescent Protein (BFP). qPCR analysis confirmed that SBPase cells were expressing SBPase at 3.2% of ActB expression and BFP cells had no measurable SBPase expression. Triplicate 70% confluent 60 mm dishes were grown for 3 hours with [6-^13^C_1_]glucose media, then metabolites were extracted, quantified, and analyzed (Fig 6A). In BFP expressing cells, S7P was 15% unlabeled, 85% M+1 labeled. In SBPase expressing cells S7P was 22% unlabeled, 72% M+1 labeled and 6% M+2 labeled. The portion of +2 labeled S7P in SBPase expressing cells was small but significant, indicating that TGME49_235700 has SBPase activity.

While the [6-^13^C_1_]glucose labeling experiment strongly supports the classification of TGME49_235700 as an SBPase, an *in vitro* assay was needed to confirm SBPase activity. SBP was not commercially available so we synthesized SBP by reacting erythrose-4-phosphate and dihydroxyacetone-phosphate with fructose bisphosphate aldolase and checked for product formation by HPLC-MS (S5 Fig). We reacted the synthesized SBP with lysate from the BFP and SBPase expressing HeLa cells, along with a no lysate control for spontaneous dephosphorylation of SBP to S7P (Fig 6B, S6 Fig). Reactions were quenched, metabolites extracted and abundance values determined via mass spectrometry. Under every condition lysate from SBPase expressing cells significantly increased S7P abundance compared to the no lysate control, while treatment with BFP cell lysate did not significantly increased S7P abundance (Fig 6B). While the crude lysate contains many protein and metabolites, including transketolase and transaldolase enzymes which react with S7P, our findings show a clear differentiation between the lysates. These findings indicate that TGME49_235700 has functional SBPase activity.

### Deletion of TGME49_235700 and [6-^13^C_1_]glucose labeling shows sedoheptulose bisphosphatase activity

To determine the role SBPase plays in *T*. *gondii* we attempted to delete the gene in type II ME49 parasites using CRISPR/Cas-9 and drug resistance selection for homologous recombination. Multiple attempts using drug selection yielded no viable knockout parasites. We hypothesized that deletion of SBPase (ΔSBPase) reduced the fitness of *T*. *gondii* and our inability to isolate a knockout was due to non-mutant parasites outcompeting the ΔSBPase parasites. We then attempted a rapid selection strategy using Fluorescence Activated Cell Sorting (FACS) for mCherry positive and GFP negative parasites. Untargeted CRISPR/Cas-9 plasmid was used as a negative control. Three independent transformations were sorted; SBPase targeted CRISPR/Cas-9, untargeted CRISPR/Cas-9 and an equal mix of SBPase and untargeted CRISPR/Cas-9. The SBPase targeted transformation generated viable mCherry expressing putative ΔSBPase parasites, the untargeted transformation yielded no mCherry parasites, and the mixed population had half as many mCherry parasites as the SBPase targeted (S7 Fig). Putative ΔSBPase parasites were cloned, but by nine days post-transformation, only a few viable plaques grew while most wells contained spacious vacuoles with non-viable parasites.

**Fig 7:**
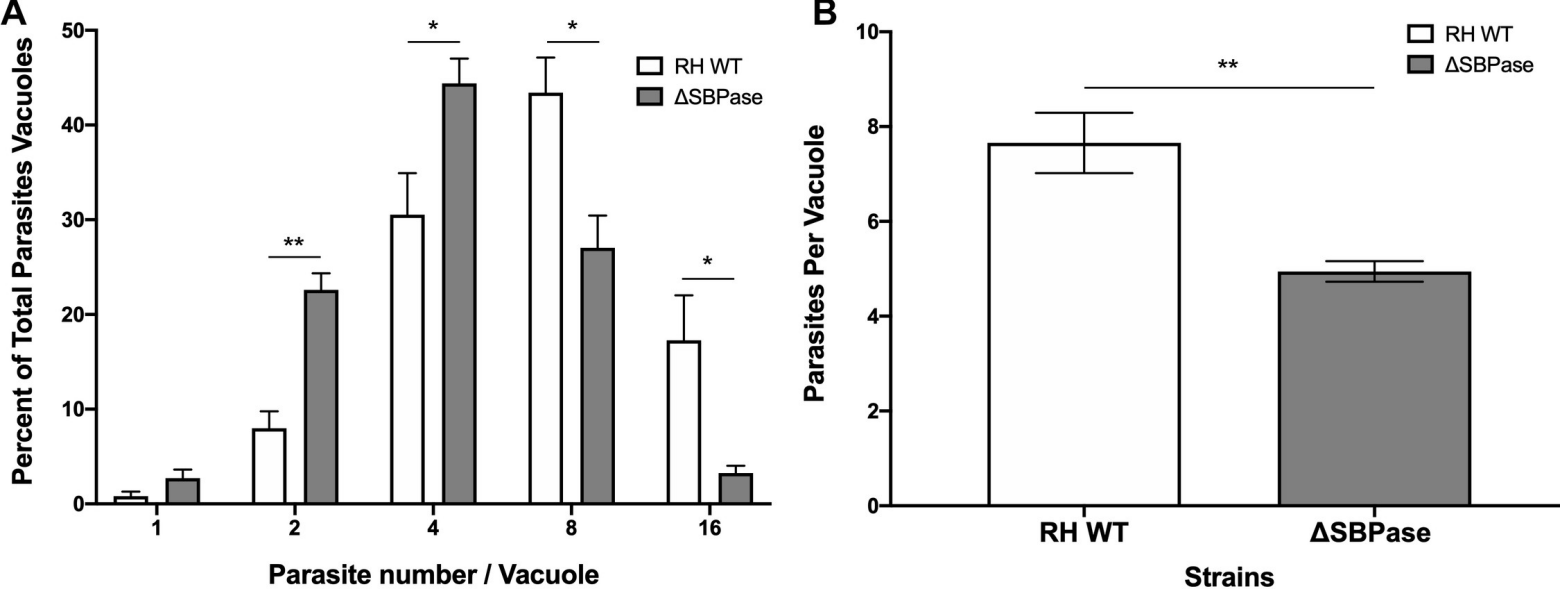
ΔSBPase parasites have a reduced replication rate. (A) The number of vacuoles containing either 1, 2, 4, 8 or 16 wild type RH (RH WT, white bars) or ΔSBPase (grey bars) parasites was counted at 24 hours post infection. Slides were blinded to reduce bias and at least 100 parasite containing vacuoles were counted per slide. Two independent experiments in duplicate were performed. Asterisks mark significant differences in abundance (*p<0.05, **p<0.01 two tailed t-test). (B) The number of parasites per vacuole was averaged for the slides counted in panel A, with wild type RH (RH WT, white bars) or ΔSBPase (grey bars). Asterisks mark significant differences in abundance (** p<0.01 two tailed t-test).

Because this gene was not predicted to be essential in type I parasites [41], we decided to generate ΔSBPase parasites in a RH type I background. Using the rapid selection strategy described above, we obtained ΔSBPase parasites and verified the deletion by PCR and Southern blot (S8 Fig). ΔSBPase parasites appeared to replicate more slowly than the wild type (WT) RH parental strain, so we quantified their replication rate. We infected HFFs with either RH WT or ΔSBPase parasites and counted the number of vacuoles containing either 1, 2, 4, 8 or 16 parasites at 12 and 24 hours post infection. At 12 hours post infection, there was no significant difference in the replication rate with RH WT having an average of 1.50 parasites per vacuole and ΔSBPase having an average of 1.47 parasites per vacuole. At 24 hours post infection, the majority of the ΔSBPase vacuoles contained 4 parasites, whereas the majority of RH WT vacuoles contained 8 parasites (Fig 7A). When the number of parasites per vacuole was averaged for the slides, RH WT infected vacuoles contained 7.7 parasites per vacuole while the ΔSBPase contained 4.9 (Fig 7B).

**Fig 8:**
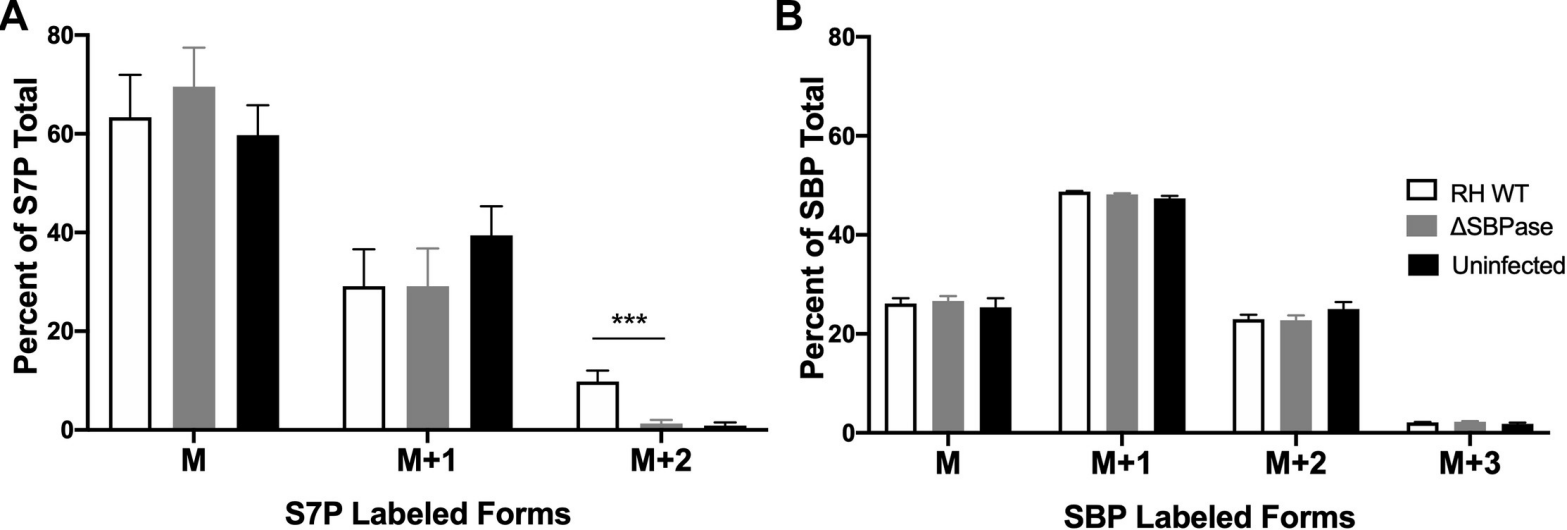
[6-^13^C_1_]glucose labeling shows that ΔSBPase parasites have reduced SBPase activity. (A) Mass (M) of S7P as a percentage of the total in HFF cells infected with wild type RH (RH WT, white bars) or ΔSBPase parasites (grey bars), or cells left uninfected (black bars). M+1 is S7P containing one ^13^C and M+2 is S7P containing two ^13^C. Asterisks mark significant differences between RH WT and the ΔSBPase for M+2 is S7P (***p<0.001 two tailed t-test). Shown in the mean with the SEM of four independent experiments, each performed in triplicate, n = 12. Total S7P ion counts in WT RH infected cells were 50135 ± 26721, in ΔSBPase infected cells there were 39904 ± 17589 S7P ion counts and in uninfected the S7P ion counts were 23960 ± 16350. (B) Mass (M) of SBP as a percentage of the total in HFF cells infected with wild type RH (white bars) or ΔSBPase parasites (grey bars), or cells left uninfected (black bars). M+1 is SBP containing one ^13^C and M+2 is SBP containing two ^13^C. No significant differences were seen between any of the SBP samples. Shown in the mean with the SEM of four independent experiments, each performed in triplicate, n = 12. Total SBP ion counts in WT RH infected cells were 1735302 ± 773808, in ΔSBPase infected cells there was 1452467 ± 792903 and in uninfected samples the SBP ion counts were 352738 ± 161284.

We then assayed for SBPase activity in the ΔSBPase parasites using [6-^13^C_1_]glucose labeling. Because the level of M+2 labeled S7P is likely proportional to the total number of parasites, we changed the assay time from 24 to 12 hours post infection as there was no difference in the parasites per vacuole between RH WT and ΔSBPase at 12 hours post infection. Triplicate dishes infected with RH WT or ΔSBPase parasites, or uninfected controls were grown for 12 hours with [6-^13^C_1_]glucose media. There was significantly more M+2 labeled S7P in RH WT parasites compared to ΔSBPase (Figs 8A and S9). The low level of M+2 labeled S7P in ΔSBPase parasites in some experiments likely comes from the two fructose 1,6-bisphosphatases (FBPase) in *T*. *gondii* (TGME49_205380 and TGME49_247510) as they have a high degree of homology (S10 Fig) with SBPase and the FBPase from cyanobacterium has been shown to be a bifunctional FBPase/SBPase [42]. *T*. *gondii* FBPase II is especially appealing as a candidate for the bifunctional activity because it contains five of the six SBP residues predicted to bind metal and both of the amino acids predicted for SBP to bind substrate (S3 Fig). The reduction in +2 labeled S7P in the ΔSBPase infected and uninfected cells was not due to a lack of +2 labeled SBP (Fig 8B).

### Overexpression of *T*. *gondii* SBPase alters infection metabolome

**Fig 9:**
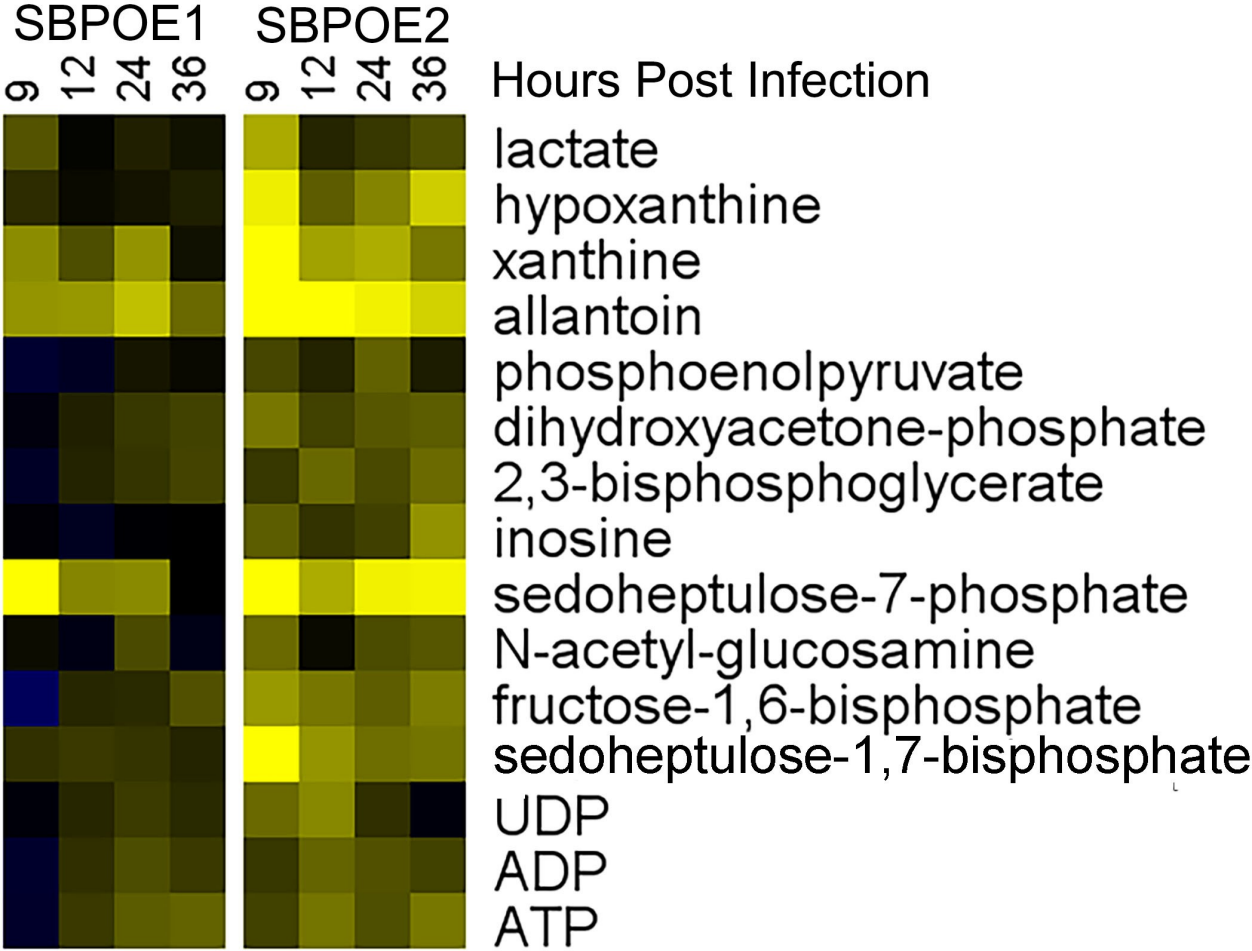
Overexpression of *T*. *gondii* SBPase Changes Infection Metabolome. Heatmaps show metabolite abundances from *T*. *gondii* infection time course of 9, 12, 24 and 36 HPI with the same scale of previous heatmaps. Duplicate samples of SBPOE1 (left) or SBPOE2 (right) infection were measured by HPLC-MS for metabolites. Metabolite abundances were averaged and normalized to the average duplicate WT abundance then log base 2 transformed (Log_2_(SBPOE Abundance/WT Abundance)). SBPOE1 and SBPOE2 differ from WT in the same areas of metabolism although changes in SBPOE2 metabolite abundance are of greater amplitude than SBPOE1.

The role of SBPase in other organisms has been well characterized [19,38,39,43, 44], but to address the role of SBPase in *T*. *gondii* metabolism, we expressed TGME49_235700 from the α-tubulin promoter and examined the infection metabolome. This construct was randomly inserted into the *T*. *gondii* genome by electroporation. Two unique overexpression clones, SBPOE1 and SBPOE2, were confirmed by Southern blot (S11 Fig). A limited metabolomics time course was carried out using WT ME49, SBPOE1 and SBPOE2, with samples taken at 9, 12, 24, and 36 HPI. The metabolomes of each SBPOE clone were normalized to the WT. While the two clones displayed similar metabolomic shifts, the amplitude of these changes was much greater in SBPOE2 than SBPOE1 (Fig 9). qPCR analysis found WT SBPase expression was 59% of Tub1A, while SBPOE1/Tub1A was 180% and SBPOE2 /Tub1A was 400% (S2 Table). Thus, the qPCR data confirmed that SBPOE2 expresses SBPase at a higher level than SBPOE1, although both express SBPase at above WT levels. S7P was 2.1 and 3 fold more abundant at 9 HPI in SBPOE1 and SBPOE2 infected populations, respectively. Multiple nucleotide metabolites increased during SBPOE1 and SBPOE2 infection over WT levels including xanthine, hypoxanthine, UDP, ADP, ATP, inosine, and allantoin. Lactate and fructose-1,6-bisphosphate abundance was also moderately increased in SBPOE1 and SBPOE2. These results indicate that SBPase plays a similar role in *S*. *cerevisiae* and *T*. *gondii* metabolism, driving the synthesis of S7P which can then flow into ribose and nucleotide synthesis.

## Discussion

Recent *T*. *gondii* studies have expanded our understanding of the flexible nature of parasite metabolism [2,7,15,16]. While most studies have exclusively examined parasite metabolism, our analysis included the host metabolome, which plays such a critical role during infection. Our study widens the scope of *T*. *gondii* metabolomics research to include the host metabolism and pairs this dual metabolome with transcriptomic analysis to identify changes in the transcription of metabolic enzymes that correlate with changes in metabolite abundance. While we expect *T*. *gondii* metabolism to make up a greater proportion of the infection metabolome as the infection progresses, multiple metabolic shifts occur prior to parasite replication. The abundance of 12 metabolites does not directly correlate to the number of parasites as parasite replication does not begin to exponentially increase until after 24 HPI (Fig 2). These metabolites were particularly intriguing to us because they may derive from parasite manipulation of the host cell metabolism. Overall, we show that *T*. *gondii* infection changes amino acid synthesis, nucleotide metabolism, glycolysis, the TCA cycle and the PPP. Increases TCA cycle metabolite abundance correlated with increased transcription of *T*. *gondii* TCA enzymes while increased PPP intermediate abundance mirrored increases in PPP enzyme expression in the host and the parasite. Changes in the PPP led to the discovery of a novel parasite enzyme, sedoheptulose bisphosphatase, which drives carbon into the non-oxidative PPP where it can be converted into ribose-5-phosphate.

Nucleotide intermediates were broadly more abundant in the infection metabolome, including nucleotides, nucleosides and the essential building block PRPP. *T*. *gondii* can synthesize pyrimidines but is a purine auxotroph. Host nucleotide metabolism was transcriptionally activated during infection, and key enzymes in *T*. *gondii* salvage pathways including adenosine kinase (AK) and hypoxanthine phosphoribosyltransferase (*HXGPRT*) were highly expressed. *T*. *gondii* putative nucleotide synthesis genes, including phosphoribosyl pyrophosphate synthase (PRS1), were also highly transcribed during infection. These results support a model in which *T*. *gondii* infection alters host nucleotide metabolism, while the parasite is actively synthesizing and scavenging nucleotide metabolites. Further experiments examining enzyme abundance and post translational modifications are needed to complete our understanding of this complex but important area of the infection metabolome.

Recent publications have greatly expanded our understanding of the *T*. *gondii* TCA cycle beyond its role in energy production [2,34]. These findings include that the mitochondrial source of acetyl-CoA is from BCKDH [34] and that a GABA shunt allows *T*. *gondii* to use glutamine to fuel its TCA cycle [2]. Also, *T*. *gondii* isocitrate dehydrogenase, which catalyzes the rate limiting step of the TCA cycle, appears to be not NAD+ dependent as the human enzyme is but instead NADP+ dependent [31]. Multiple TCA intermediates were more abundant late in infection when parasite numbers were greatest. Moreover, *T*. *gondii* TCA cycle enzymes were highly transcribed throughout infection, while expression of almost all of host TCA enzymes remained at or below the levels of uninfected cells. These findings support prior work showing the importance of the *T*. *gondii* TCA cycle, while the lack of change in host enzyme expression coupled with observations of host mitochondrial fragmentation indicate that the host TCA cycle may not be critical during infection [2,45].

In contrast to the *T*. *gondii* TCA cycle, little research has investigated the PPP. The PPP has oxidative and non-oxidative halves and produces ribose-5-phosphate and NADPH. The energetically driven oxidative pathway synthesizes NADPH and funnels carbon from glucose-6-phosphate into the pentose monophosphate pool. From that pool, carbon can either flow into biosynthetic or the non-oxidative pathways. The non-oxidative PPP is catalyzed by transketolase and transaldolase, and is a bidirectional connection between the pentose and hexose monophosphate pools. Previous work has demonstrated that *T*. *gondii* has an active oxidative PPP [2,5,6] and has annotated genes for the transketolase and transaldolase enzymes of the non-oxidative PPP [46]. Metabolomics studies using purified parasites have observed PPP intermediates and demonstrated that there is active flux of carbon through the pathway [2,5]. Intermediates in the oxidative and non-oxidative pathways were more abundant in the infection metabolome. Host oxidative PPP genes are highly upregulated during infection, while non-oxidative genes are consistently expressed. The lack of identified oxidative PPP genes in *T*. *gondii* complicates analysis but there was high expression of putative oxidative and non-oxidative PPP genes throughout infection. The infection PPP warrants further study, given the uncharacterized *T*. *gondii* oxidative pathway enzymes and the unknown cause of increased host oxidative PPP enzyme transcription.

Increases in SBP and OBP abundance, followed by increases in S7P and O8P, led us to search for a link between these metabolites. SBPase was the most likely candidate as it catalyzes the production of S7P and O8P from SBP and OBP, respectively. SBPase activity pulls carbon from glycolysis into S7P in the non-oxidative PPP, where it then can be converted into ribose-5-phosphate. SBPase activity creates an energetically driven non-oxidative PPP by the dephosphorylation of SBP, which may be advantageous to *T*. *gondii* as it’s TCA cycle appears to be NADP+ dependent [24]. The potential redox imbalance for ribose synthesis set up by *T*. *gondii’s* TCA cycle and oxidative PPP both being NADP+ dependent is likely settled by SPBase activity as well as the transaldolase and transketolase enzymes being reversible.

While mammals do not appear to have SBPase activity [19–21], *T*. *gondii* has a putative SBPase (TGME49_235700). Using a previously published [6-^13^C_1_]glucose labeling method, we assayed for SBPase activity. Cells lacking SBPase will generate unlabeled or +1 ^13^C S7P while SBPase produces a pool of +2 S7P (diagramed in S4 Fig). This simple model is complicated by a highly active futile cycle in *T*. *gondii* glycolysis, which could result in S7P double labeling from double labeled F6P. Using [6-^13^C_1_]glucose media, we compared the isotopic composition of S7P in infected and uninfected cells. S7P from uninfected cells was either +1 or unlabeled as expected while infected cells had a large pool of +2 S7P, indicating possible SBPase activity. The portion of double labeled F6P (3.4%) was over 3 times smaller than the portion of double labeled S7P (14%). If futile cycling alone was responsible for double labeling of S7P these percentages would be expected to be similar. The higher portion of double labeled S7P indicated that there was another source of this labeling, likely via an active *T*. *gondii* SBPase.

To examine SBPase activity, we used a lentiviral system to express TGME49_235700 in HeLa cells and measured SBPase activity with the labeling assay. BFP negative control cells had no significant +2 S7P while cells expressing putative SBPase had a significant +2 S7P pool after only 3 hours of labeling. The dramatic reduction SBP pools in the SBPase expressing cells (only 461 ion counts ± 368) contrasted to the sizable SBP pool in BFP expressing cells (254077 ion counts ± 117590) further supports the identification of TGME49_235700 as a SBPase. We also performed an *in vitro* SBPase activity assay using lysate from SBPase and BFP expressing cells. Lysate from SBPase expressing cells generated substantial and significant increased S7P abundance compared to the BFP expressing cells and no lysate controls. These findings show that TGME49_235700 codes for a functional SBPase. Further studies are warranted to determine the kinetics and specificity of the *T*. *gondii* SBPase and to examine its relevance to other stages of the *T*. *gondii* life cycle.

To determine the role of the SBPase in *T*. *gondii* metabolism the gene was overexpressed from the α-tubulin promoter. By examining the infection metabolome of two SBPase overexpressing strains (SPBOE1 and SBPOE2) compared to a wild type, we determined the impact of SBPase overexpression on the infection metabolome. SBPase overexpression led to increased abundance in S7P, several nucleotide metabolites and glycolytic intermediates. These findings strongly indicate that *T*. *gondii* SBPase drives carbon into S7P where it can flow into either ribose-5-phosphate and nucleotide metabolism or down the non-oxidative PPP and back into glycolysis. These changes were more pronounced in SBPOE2 than SBPOE1, which was supported by qPCR data showing higher levels of SBPase expression in SBPOE2 than SBPOE1. While our data does not give us information regarding substrate specificity, *S*. *cerevisiae* SBPase possessed *in vitro* FBPase activity but not *in vivo* FBPase activity [19]. While we do see an increase in fructose-1,6-bisphosphate during SBPOE infection we see no change in the abundance of F6P, indicating that *T*. *gondii* SBPase may be similarly substrate specific for SBP. The increase in SBP abundance early during SBPOE1 and SBPOE2 infection was surprising. This increase in SBP may be due to synthesis by fructose bisphosphate aldolase, driven by an initial depletion of SBP by SBPase. Two other unexpected changes were the increase in xanthine and hypoxanthine abundance, given that *T*. *gondii* is incapable of synthesizing those metabolites and must scavenge them from the host. It is possible that the changes in metabolism caused by SBPase overexpression resulted in increased parasite nucleotide scavenging from the host cells, which in turn changed host nucleotide metabolism.

Together these results demonstrate that SBPase catalyzes a novel energetically favorable pathway for pushing carbon into the non-oxidative PPP where it is available for ribose-5-phosphate synthesis. SBPase likely plays an important role in regulating *T*. *gondii* ribose synthesis, acting as a switch to funnel carbon from glycolysis into ribose synthesis. Further study is needed of *T*. *gondii’s* SBPase and its putative orthologs in *Trypanosoma* and *Neospora* to determine if these are true orthologs and their role in parasite metabolism [38,47].

## Methods

### *T*. *gondii* strains and cell culture

Low passage type II ME49 *T*. *gondii* used in all experiments, except for the SBPase deletion experiments, which were performed in RH type I parasites. Human Foreskin Fibroblasts (HFFs) from the ATCC were grown in 60mm dishes in DMEM with 10% Fetal Bovine Serum (FBS), 2 mM L-glutamine, and 1% penicillin-streptomycin (Sigma-Aldrich). Once HFFs were in deep quiescence, defined as 10 days post confluency, DMEM media was changed to metabolomic media, RPMI1640 supplemented with 2 mM L-glutamine, 1% FBS dialyzed against PBS (MW cutoff of 10 kD), 10mM HEPES, and 1% penicillin-streptomycin. After 35 hours, the media was again changed with metabolic media, 1 hour before infection with *T*. *gondii*.

### Infection time course metabolomics

HFF dishes in triplicate were infected with 2 X 10^6^ tachyzoites, or mock infected with an equal addition of media by volume. This resulted in an MOI of 0.625, with approximately 3.2 X 10^6^ fibroblasts per dish. The MOI was chosen to result in a high level of infection (estimated 40%) while minimizing multiple infections of the same host cell, which could result in sample loss due to early lysis. At time points 1.5, 3, 6, 9, 12, 24, 36, and 48 hours post infection (HPI), dishes were washed 3x with ice cold PBS, then quenched with 80:20 HPLC grade Methanol:Water (Sigma-Aldrich). Dishes were incubated on dry ice in a -80°C for 15 minutes. Plates were scraped, the solution removed and spun at 2500 x g for 5 minutes at 4°C. The supernatant was removed and stored on ice, then the pellet was washed again in quenching solution and re-spun. Supernatants were combined, dried down under a N_2_ gas manifold, and stored at -80°C.

Samples were resuspended in 150 μL HPLC grade water (Fisher Optima) for analysis on a Thermo-Fisher Vanquish Horizon UHPLC joined by electrospray ionization (negative mode) to a hybrid quadrupole-Orbitrap high resolution mass spectrometer (Q Exactive Orbitrap; Thermo Scientific). Chromatography was performed using a 100 mm X 2.1 mm X 1.7 μm BEH C18 column (Acquity) at 30°C. 15 μL of sample was injected via an autosampler at 4°C and flow rate was 200 μL/minute. Solvent A was 97:3 water/methanol with 9 mM Acetate and 10 mM tributylamine (TBA) with a pH of 8.2 (Sigma-Aldrich). Solvent B was 100% methanol with no TBA (Sigma-Aldrich). Products were eluted in 95% A/5% B for 2.5 minutes, then a gradient of 95% A/5% B to 5% A/95% B over 14.5 minutes, then held for an additional 2.5 minutes at 5%A/95%B. The gradient was returned to 95% A/5% B over 0.5 minutes, and held for 5 minutes to re-equilibrate the column. Peaks were matched to known standards for identification [20,21], except for S7P (Sigma-Aldrich) and SBP which was produced for this study (S5 Fig). Data analysis was performed using the Metabolomics Analysis and Visualization Engine (MAVEN) software [48]. Heat maps were generated using the Multi Experiment Viewer program, and the values represented in the heat map are shown in S3 Table. All RAW files generated in this paper may be found in the online repository Metabolights, study number 899 (MTBLS899).

### Immunofluorescence Assay for *T*. *gondii* Replication

To determine the average number of parasites per vacuole throughout infection, HFFs with glass coverslips were infected with either ME49, RH WT or ΔSBPase at the same MOI as the metabolomics experiments. At each time point, coverslips were rinsed with PBS, fixed with 3% formaldehyde, permeabilized with 0.3% Tween 20, incubated with 1:500 mouse chronic infection sera and 1:1000 goat anti-mouse Alexa Fluor 594, then mounted with DAPI to allow for the counting of parasite nuclei and mounted on a slide. At least 100 infected cells were counted from each time point and the average number of *T*. *gondii* per infected cell was calculated.

### RNAseq

Samples from uninfected or infected 60 mm dishes of HFFs paired to infected metabolomics dishes were resuspended in TRIzol. RNA was extracted per the manufacturer’s protocol, assayed for quality with an Agilent Bioanalyzer and Nano-6000 RNA chip, and synthesized into libraries with a TruSeq RNA Library Preparation Kit v2. Sequencing was performed through the UW Biotechnology Sequencing Center, using Illumina HiSeq2000. Unfortunately the 3 HPI sample was compromised during library preparation and therefore there is no expression data for that time point. The accession number for this data is PRJNA497277 through the NCBI Sequence Read Archive. All *T*. *gondii* gene accession numbers can be found on ToxoDB.org.

### Read mapping and analysis

Approximately 370 million 150 base pair reads were generated from the 9 samples and uploaded to the online Galaxy platform. Reads were filtered using the FASTQ Groomer (version 1.0.4) and then aligned to the *T*. *gondii* and human genomes (TGME49 version 2013-04-23, Human version 2013-12-24 HG38) using Tophat2 (version 2.0.14), with the parameters as follows: Max edit distance 2, read mismatch 2, anchor length 8, minimum intron length 70, maximum intron length 500000, max insertion and deletion length 3, number of mismatches allowed 2, and minimum length of read segments 25. Read filtering and mapping are summarized in Table 1. Alignments were converted into FPKM expression values using the Cufflinks Cuffnorm tool and differential changes calculated using Cuffdiff (version 2.2.1.0).

### Taurine metabolism assay

HFFs were grown to deep quiescence in 60 mm dishes, then the media was changed to metabolic media for 35 hours. One hour before infection, the media was change to metabolomic media supplemented with 2 mM ^15^N Taurine (Sigma-Aldrich). Dishes in triplicate were infected with 2 X 10^6^ tachyzoites and metabolites were extracted at 3, 6, 9, 12, and 24 HPI as previously described. Metabolites were extracted from a single time point of triplicate mock-infected dishes at 1.5 HPI to measure taurine uptake from the media. MAVEN software was used to identify metabolite incorporation of ^15^N from taurine metabolism.

### Media taurine assay

HFFs were grown to deep quiescence in 60 mm dishes, then the media was changed to metabolic media for 35 hours. One hour before infection, the media was change to one of two metabolomic medias: no taurine or 44μM taurine (Sigma-Aldrich). Dishes were infected with 2 X 10^6^ tachyzoites or mock-infected in triplicate for each media condition. Media samples were taken and metabolites were extracted at 0, 1.5, 3, 6, 9, 12, 24, 36, and 48 HPI. Extraction was performed by adding 80:20 HPLC grade Methanol:Water quenching solution directly to media, incubating samples in a -80°C for 15 minutes and then proceeding with extraction at the first centrifugation step. MAVEN software was used to measure media taurine abundance in each condition over the course of infection.

### Sedoheptulose bisphosphatase activity assay

HFFs were grown to deep quiescence in 60 mm dishes as before, then the media was changed to metabolic media for 35 hours. One hour before infection, the media was change to metabolomic media except using glucose free RPMI1640 supplemented with [6-^13^C_1_]glucose at normal glucose concentration (2 g/L) (Sigma-Aldrich). Dishes in triplicate were infected with 2 X 10^6^ tachyzoites, or mock infected with an equal addition of media by volume. At 12 HPI for RH type I infections or 24 HPI for ME49 type II infections, dishes were quenched and metabolites extracted, processed, and analyzed as previously described in the “infection time course metabolomics” section, except that samples were resuspended in 75 μL of HPLC grade water and 20 μL was injected. MAVEN software was used to identify the isotopic forms of sedoheptulose-7-phosphate and sedoheptulose-1,7-bisphosphate, while correcting for the natural abundance of ^13^C.

### Deletion of TGME49_235700

The deletion of TGME49_235700 was constructed by electroporation with two plasmids, the donor plasmid containing homologous flanking regions of TGME49_235700 and the Cas9 plasmid that codes the gene-specific gRNA. All the PCR amplifications were performed using Q5 DNA polymerase (New England Biolabs, M0491L) and plasmids constructed using Gibson assembly (New England Biolabs, E2611L) with pBluescript as the plasmid backbone. The homologous regions for the donor plasmid were PCR amplified using genomic DNA from the ME49 strain with primers KO US-FW and KO US-RV for amplification of the upstream region and primers KO DS-FW and KO DS-RV for amplification of the downstream region. The donor plasmid contains the mCherry gene under a *T*. *gondii* tubulin promoter in between the upstream and downstream flanking regions, and the eGFP gene under *T*. *gondii* tubulin promoter after the downstream flank. mCherry and eGFP genes were amplified using the primers KO mCherry-FW and KO mCherry-RV, and KO GFP-FW and KO GFP-RV, respectively. The pCas9/CAT plasmid [41] was mutated using the primers Cas9 FW and Cas9 RV to add the SBPase-specific gRNA. For the deletion of TGME49_235700, RH WT tachyzoites were transfected with the 100 μg of the Cas9 plasmid and the 25 μg of the linearized donor plasmid. mCherry positive and eGFP negative tachyzoites were sorted by flow cytometry. Clones were grown and genomic DNA extracted for confirmation.

The deletion of TGME49_235700 was confirmed by PCR using mCherry-3’UTR FW and mCherry-3’UTR RV, which amplified a 2.2 kb only in the deletion mutant. To confirm genomic DNA integrity, we used primers SAG-1 FW and SAG1 RV, which amplified a 250 bp band in all samples. For the Southern blot, tachyzoites genomic DNA was extracted, digested with SacI, separated in an agarose gel, transferred to a positively charged membrane (Amersham), and incubated with ^32^P-labelled probe specific to the TGME49_235700 PCR amplified with KO probe FW and KO probe RV.

### Sedoheptulose Bisphosphatase Overexpression (SBPOE) in *T*. *gondii*

The transcript of the putative *T*. *gondii* sedoheptulose bisphosphatase (TGME49_235700) was amplified from Me49 cDNA made with SuperScript III First-Strand Synthesis System for RT-PCR (Invitrogen). The product was joined with a *T*. *gondii* α-tubulin promoter via Splicing by Overlap Extension (SOE) PCR, then ligated into the dihydrofolate reductase plasmid DHFR-TS [49] using Gibson Assembly. Low pass ME49 was electroporated with 20 μg linearized plasmid and selected with 1 μM pyrimethamine. Parasites were cloned by limiting dilution and expanded for genomic DNA (gDNA) extraction. gDNA was digested with XbaI and examined by Southern Blot as previously described with a probe that bound the native SBPase and the overexpression construct. XbaI digestion yields a 5kb band for the wild type SBPase and a band of unknown length for the overexpression construct, with successful incorporation yielding a distinctive double banding pattern for each unique clone. All primers used in this study are described in S4 Table.

### Sedoheptulose bisphosphatase overexpression in HeLa cells

Open reading frame (ORF) of the putative *T*. *gondii* sedoheptulose bisphosphatase (TGME49_235700) was amplified from Me49 cDNA, cloned into pENTR Tev-D-TOPO [42], then recombined into pLX304 using Gateway cloning. The expression plasmid was transfected in 293T cells with psPAX2 and pCMV-VSV-G and lentiviruses were harvested from the supernatant 48 HPI. 75% confluent 6mm dishes of HeLa cells (ATCC) were infected with 200 μL of lentivirus mixed with 0.2 μL of 8mg/mL Polybrene. 24 HPI media was replaced with media containing 10 μg/mL blasticidin for 3 passages post clearance of transduced cells [50]. As a negative control BFP was inserted into the genome of a second population of HeLa cells using the same delivery system. Both populations were screened for SBPase activity in triplicate at 70% confluency using previously described isotope labeling methods, but without *T*. *gondii* infection and with three hours of growth in labeled media.

### SBPase lysate activity assay

SBP was produced using the following reaction conditions, adapted from a previous manuscript, incubated at 37° C for 15 minutes; base buffer of 50 mM Tris HCl pH 7.5, 2 mM MnCl_2_, 1 mM erythrose-4-phosphate (Sigma), 1 mM dihydroxyacetone-phosphate (Sigma), 1 mM PMSF, 2 units of Fructose Bisphosphate Aldolase from rabbit muscle (Sigma). Protein lysates were collected from 70% confluent 10 cm dishes of HeLa cells, which were scraped, pelleted via centrifugation at 750 x g, and rinsed with PBS. The pellet was resuspended in TrisHCl pH 7.5 with 1mM PMSF, then syringed 5 times to lyse cells. Lysates were centrifuged at 14,000 x g for 10 minutes to clear debris, then supernatant containing cytoplasmic protein was removed and protein concentration was determined via the Bradford assay. Reactions to test lysates began with 15 minutes of SBP production, then either 5 μg or 2.5 μg of lysate from HeLa cells expressing either BFP or TGME49_235700 was added to the reaction, along with a no lysate control which received an equal volume of Tris HCl pH 7.5. At 30 and 60 minutes post lysate addition reactions were quenched by the addition of 1.2 ml of 80:20 methanol:water. All conditions, including variations in lysate type, lysate abundance, and time of incubation, were performed in triplicate. All samples were incubated at -80° C for 15 minutes, before being spun at 14000 x g for 5 min to pellet protein debris. Supernatant was removed and dried down under a N_2_ gas flow. Dried down samples were resuspended with HPLC grade water and analyzed via mass spectrometry using our standard method to examine metabolite abundance. Data analysis was performed with the MAVEN software program.

### qPCR

Primers specific to *T*. *gondii* Tubulin subunit A or human actin subunit B were used as the normalization control (S4 Table). SBPase expression was compared between *T*. *gondii* strains SBPOE1, SBPOE2 and WT, and between HeLa populations with SBPOE or with BFP. cDNA was extracted from all cell types using TRIzol and converted to cDNA with SuperScript® III First-Strand Synthesis System for RT-PCR (Invitrogen). All cDNA samples were nanodropped for concentration and normalized to 100 ng/μl. qPCR was performed using iTaq Universal SYBR Green Supermix with the following cycling conditions: 98°C 30 seconds, then 40 cycles consisting of a 15 second 98°C first step and a 60 second 60°C second step. Data was processed to compare transcript abundance of the gene of interest (SBPase) to the abundance of the relevant housekeeping gene using the following equation: (E_housekeeping_^Ct^ / E_SBPase_^Ct^) X 100 [51]. Primer efficiencies were calculated using a standard dilution series and each sample Ct value was calculated by averaging two technical replicates.

## Supporting information

S1 Fig *T. gondii* infection changes the metabolome. Heatmaps show metabolite abundance over 48 hours of *T. gondii* infection in two independent experiments (Panels A and B). Infected and uninfected dishes of HFFs were metabolically quenched and metabolites were extracted at 1.5, 3, 6, 9, 12, 24, 36 and 48 Hours Post Infection. Metabolomes were quantified using HPLC-MS and metabolites were identified with known standards. Infected sample abundances were normalized to uninfected abundance then log base 2 transformed (Log2(Infected Abundance/Uninfected Abundance)) with blue being less abundant and yellow more abundant.
(TIF)

S2 Fig Taurine media abundance. Triplicate infected and uninfected media samples were taken over the 48 hour time course from dishes in standard metabolomic media (0 Taurine) or from media supplemented with 44 μM taurine. Metabolites were extracted and quantified with HPLC MS. Infection media taurine abundance was averaged and normalized to the average uninfected media abundance then log base 2 transformed.
(TIF)

S3 Fig SBPase alignment. *T*. *gondii* SBPase (Tg) predicted amino acid sequence was aligned to the sequence of *T*. *brucei* (Tb), *A*. *thaliana* (At) and *S*. *cerevisiae* (SC) SBPases using the T-Coffee program [44] and presented using ExPASy BoxShade (https://embnet.vital-it.ch/software/BOX_form.html). The *T*. *brucei* gene ID is Tb927.2.5800, the *A*. *thaliana* gene ID At3g55800 and the *S*. *cerevisiae* gene ID is YKR043C. Residues of the *A*. *thaliana* SBP predicted for metal binding are boxed in blue and substrate binding are boxed in red (https://www.uniprot.org/uniprot/P46283).
(TIF)

S4 Fig [6-^13^C_1_]glucose labeling. Schematic of the PPP detailed in Fig 4. Simplified diagram labeling assay previously developed (19) shows the potential metabolism of glucose labeled with ^13^C at the sixth carbon. Each circle is a carbon atom, and all molecules have open circles indicating unlabeled carbons and black circles indicating ^13^C labeled carbons. Some molecules have red circles, indicating a potential second ^13^C label. Enzyme catalyzed reactions are indicated with either double or single ended arrows to indicate directionality, with the red arrow indicating the proposed new activity catalyzed by SBPase. Abbreviations are as follows: glucose-6-phosphate (G6P), fructose-6-phosphate (F6P), fructose-1,6-bisphosphate (FBP), glyceraldehyde-3-phosphate (GAP), dihydroxyacetone-phosphate (DHAP), erythrose-4-phosphate (E4P), sedoheptulose-1,7-bisphosphate (SBP), sedoheptulose-7-phosphate (S7P), ribose-5-phosphate (Ri5P), xylulose-5-phosphate (X5P), and ribulose-5-phosphate (Ru5P).
(TIF)

S5 Fig Spectrum of synthesized SBP. SBP was synthesized by reacting erythrose-4-phosphate and dihydroxyacetone-phosphate with fructose bisphosphate aldolase and product formation was checked by HPLC-MS in negative ionization mode. A dominate peak was seen at 369 kDa, corresponding to the molecular mass of SBP. A minor peak was seen at 370 kDa, likely corresponding to non- or alternatively ionized SBP. The bottom panel is a close-up of these two peaks from the top panel.
(TIF)

S6 Fig Expression of *T*. *gondii* SBPase in HeLa results in SBPase activity. SBP was synthesized from erythrose-4-phosphate, dihydroxyacetone phosphate, and fructose bisphosphate aldolase for 15 minutes. S7P abundance was measured in triplicate after incubation with either no lysate (grey circles), BFP expressing HeLa cell lysate (black shapes), or SBPase expressing HeLa cell lysate (white shapes). Incubation was carried out with either 2.5 μg (squares) or 5 μg (triangles) lysate for 60 minutes. Error bars mark mean and a 95% confidence interval.
(TIF)

S7 Fig FACS analysis of attempted SBPase knockout with mCherry sort. ME49 parasites were electroporated with an mCherry positive selectable marker and one of three CRISPR/cas-9 plasmids; SBPase targeted CRISPR/Cas-9 (Targeted), untargeted CRISPR/Cas-9 (Untargeted), and an equal mix of SBPase and untargeted CRISPR/Cas-9 (Mixed). Electroporated parasites recovered and grew for 72 hours before FACS sorting and cloning. The mixed CRISPR population had half as many mCherry positive parasites as the SBPase targeted population and the untargeted CRISPR population had no mCherry expressing parasites.
(TIF)

S8 Fig Confirmation of TGME49_235700 deletion (ΔSBPase). (A) The reaction used mCherry-3’UTR FW and mCherry-3’UTR RV with genomic DNA and reactions were run using a temperature gradient from 55 to 65°C. Expect band size is 2.2 kb only in ΔSBPase. DNA markers in the far-left lane are the 1 kb Plus (Thermo) and the arrowhead shows the 1.5 kb band. (B) SAG-1 FW and SAG1 RV primers used as control of the genomic DNA quality and reactions were run using a temperature gradient from 55 to 65°C. Expect band size is 250 bp in all lanes and DNA markers in the far-left lane are the 1 kb Plus (Thermo) and the arrowhead shows the 1.5 kb band. (C) Genomic DNA from wild type RH (WT) or ΔSBPase parasites was extracted, digested with SacI, separated in an agarose gel, transferred to a positively charged membrane (Amersham), and incubated with ^32^P-labelled probe specific to the TGME49_235700 downstream region. The arrow indicates the 8 kb band expected for WT parasites; the arrowhead shows the 1.5 kb band expected for ΔSBPase parasites.
(TIF)

S9 Fig Independent experiment using [6-^13^C_1_]glucose labeling to show that ΔSBPase parasites do not have SBPase activity. Mass (M) of S7P as a percentage of the total in HFF cells infected with wild type RH (RH WT, white bars) or ΔSBPase parasites (grey bars), or cells left uninfected (black bars). M+1 is S7P containing one ^13^C and M+2 is S7P containing two ^13^C. Error bars are a 95% confidence interval. Each of the four independent experiments was performed in triplicate.
(TIF)

S10 Fig *T*. *gondii* SBPase and FBPases alignment. The predicted amino acid sequences from TGME49_235700 (SBPase), TGME49_205380 (FBPase I) and TGME49_247510 FBPase II were aligned using the T-Coffee program [44] and presented using ExPASy BoxShade (https://embnet.vital-it.ch/software/BOX_form.html). Residues of SBP predicted for metal binding are boxed in blue and substrate binding are boxed in red (https://www.uniprot.org/uniprot/P46283).
(TIF)

S11 Fig SBPOE1 and SBPOE2 Southern blot. Southern blotting identified two unique SBPOE clones. Genomic DNA was extracted from clonal populations of *T*. *gondii*, digested with restriction enzyme XbaI and assayed via Southern blot with an SBPase targeted probe. XbaI digestion yields an expected wild type SBPase band of 5 kb, while the overexpression inserts should yield a second band of unknown length depending on where the overexpression clone was inserted into the genome. A 5 kb WT band was present in all populations and a secondary band was observed in SBPOE1 (S1) and SBPOE2 (S2), indicating a second SBPase gene insertion. The secondary band sizes were different in SBPOE1 and SBPOE2, showing they are unique clones. A white arrow indicates the wild type band in the wild type sample, while two red arrows indicate the secondary bands in the SBPOE clones. Although the clones marked with an X had insertions, they were not used in any further experiments.
(TIF)

S1 Table Infection metabolome p-values. Metabolite abundance values were compared between triplicate infected and uninfected samples for each metabolite and at each time point over the infection time course using a two tailed t-test. Shown are the p-values of these changes for each metabolite at each time point.
(DOCX)

S2 Table qPCR Ct values table. Ct values for qPCR measuring the expression of SBPase relative to a housekeeping gene. In HeLa cells SBPase and ActB expression was measured for SBPase and BFP expressing cells. In *T*. *gondii* SBPase and Tub1A expression was measured for wild type, SBPOE1, and SBPOE2 strains. For all data triplicate biological samples were assayed, with each biological sample run with two technical replicates.
(DOCX)

S3 Table Means for metabolite abundances for Fig 1. The mean of the fold change of infected over uninfected (Log_2_) for each metabolite abundance during the infection time course from 1.5 to 48 hours post infection, with each column representing a time point. These means are graphically represented in the large heat map in Fig 1 and the p-values for each are listed in S1 Table.
(DOCX)

S4 Table Primers table. All primers are listed in 5’ to 3’ orientation, with the gene they were targeted to and the where experiment they were used.
(DOCX)
