# Supplementary figures and images for: Dual metabolomic profiling uncovers Toxoplasma manipulation of the host metabolome and the discovery of a novel parasite metabolic capability
Source: PLoS Pathog. 2020 Apr 7;16(4):e1008432. doi: 10.1371/journal.ppat.1008432 (PMC7164669; doi:10.1371/journal.ppat.1008432)

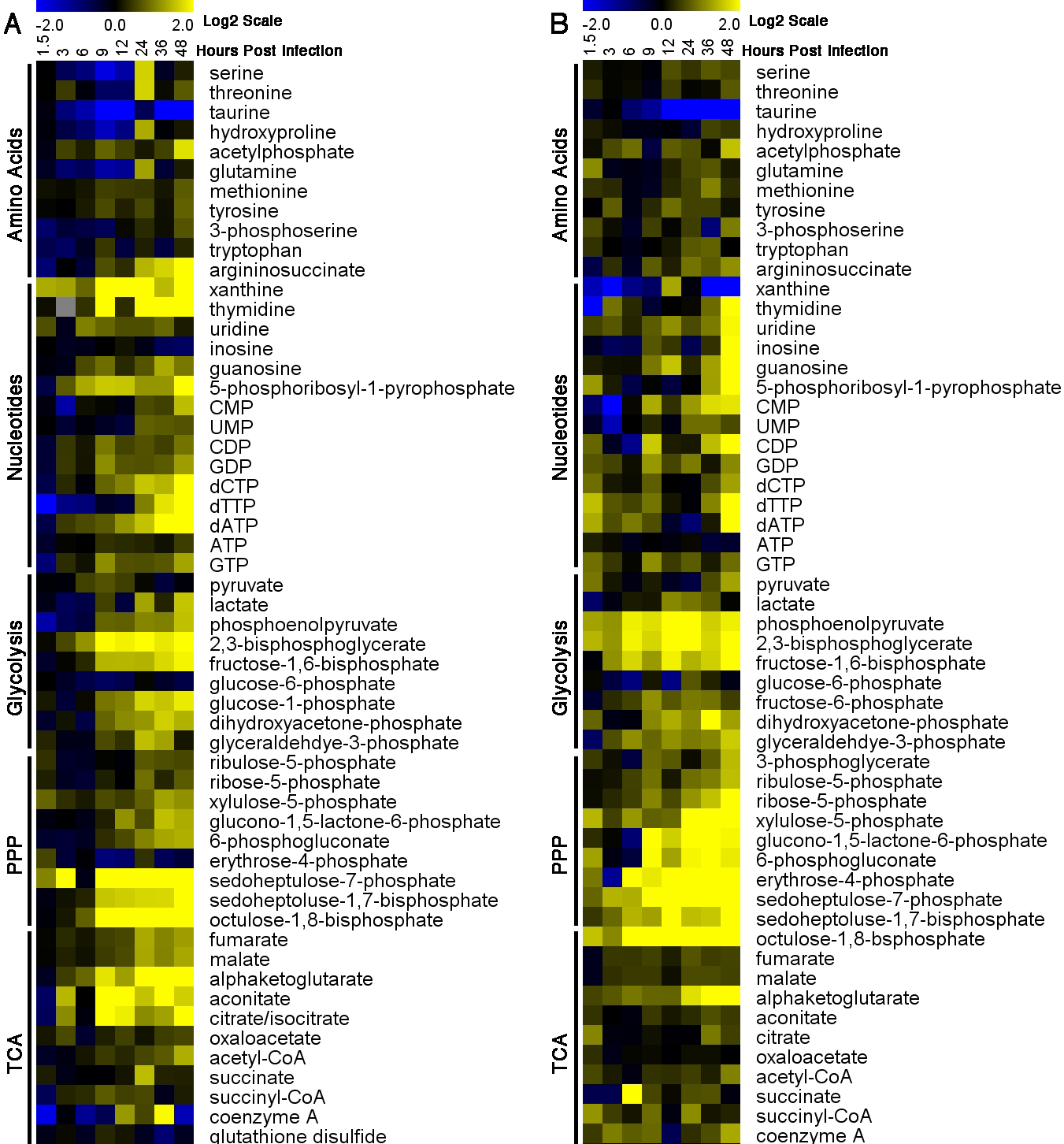

Supplement: S1 Fig — Heatmaps show metabolite abundance over 48 hours of T. gondii infection in two independent experiments (Panels A and B). Infected and uninfected dishes of HFFs were metabolically quenched and metabolites were extracted at 1.5, 3, 6, 9, 12, 24, 36 and 48 Hours Post Infection. Metabolomes were quantified using HPLC-MS and metabolites were identified with known standards. Infected sample abundances were normalized to uninfected abundance then log base 2 transformed (Log2(Infected Abundance/Uninfected Abundance)) with blue being less abundant and yellow more abundant. (TIF) [file ppat.1008432.s001.tif]

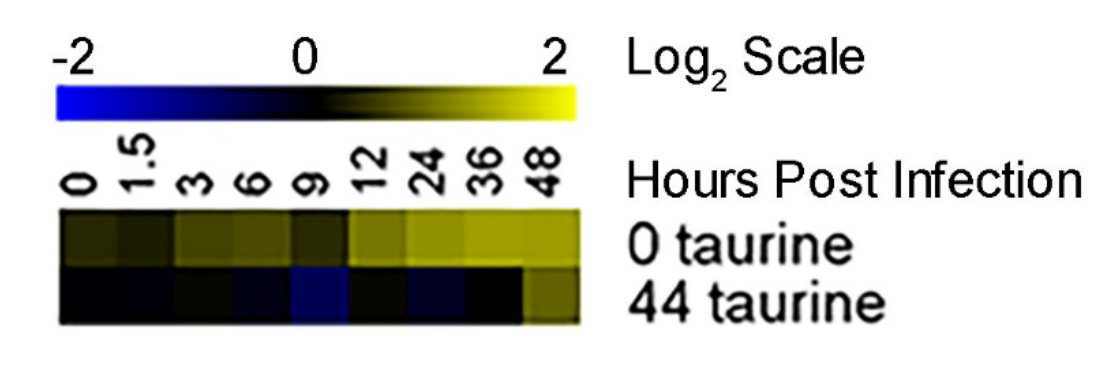

Supplement: S2 Fig — Triplicate infected and uninfected media samples were taken over the 48 hour time course from dishes in standard metabolomic media (0 Taurine) or from media supplemented with 44 μM taurine. Metabolites were extracted and quantified with HPLC MS. Infection media taurine abundance was averaged and normalized to the average uninfected media abundance then log base 2 transformed. (TIF) [file ppat.1008432.s002.tif]

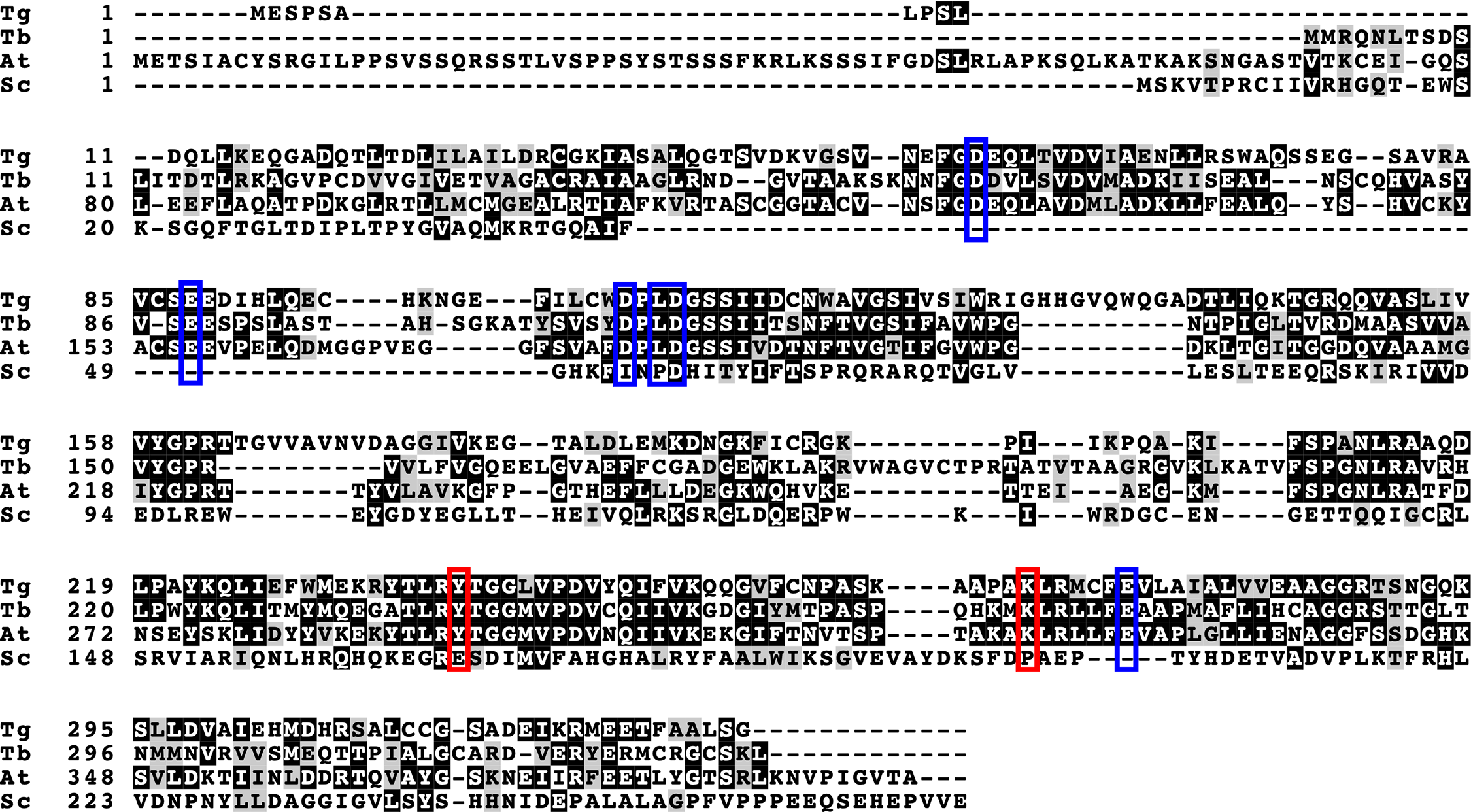

Supplement: S3 Fig — T. gondii SBPase (Tg) predicted amino acid sequence was aligned to the sequence of T. brucei (Tb), A. thaliana (At) and S. cerevisiae (SC) SBPases using the T-Coffee program [44] and presented using ExPASy BoxShade (https://embnet.vital-it.ch/software/BOX_form.html). The T. brucei gene ID is Tb927.2.5800, the A. thaliana gene ID At3g55800 and the S. cerevisiae gene ID is YKR043C. Residues of the A. thaliana SBP predicted for metal binding are boxed in blue and substrate binding are boxed in red (https://www.uniprot.org/uniprot/P46283). (TIF) [file ppat.1008432.s003.tif]

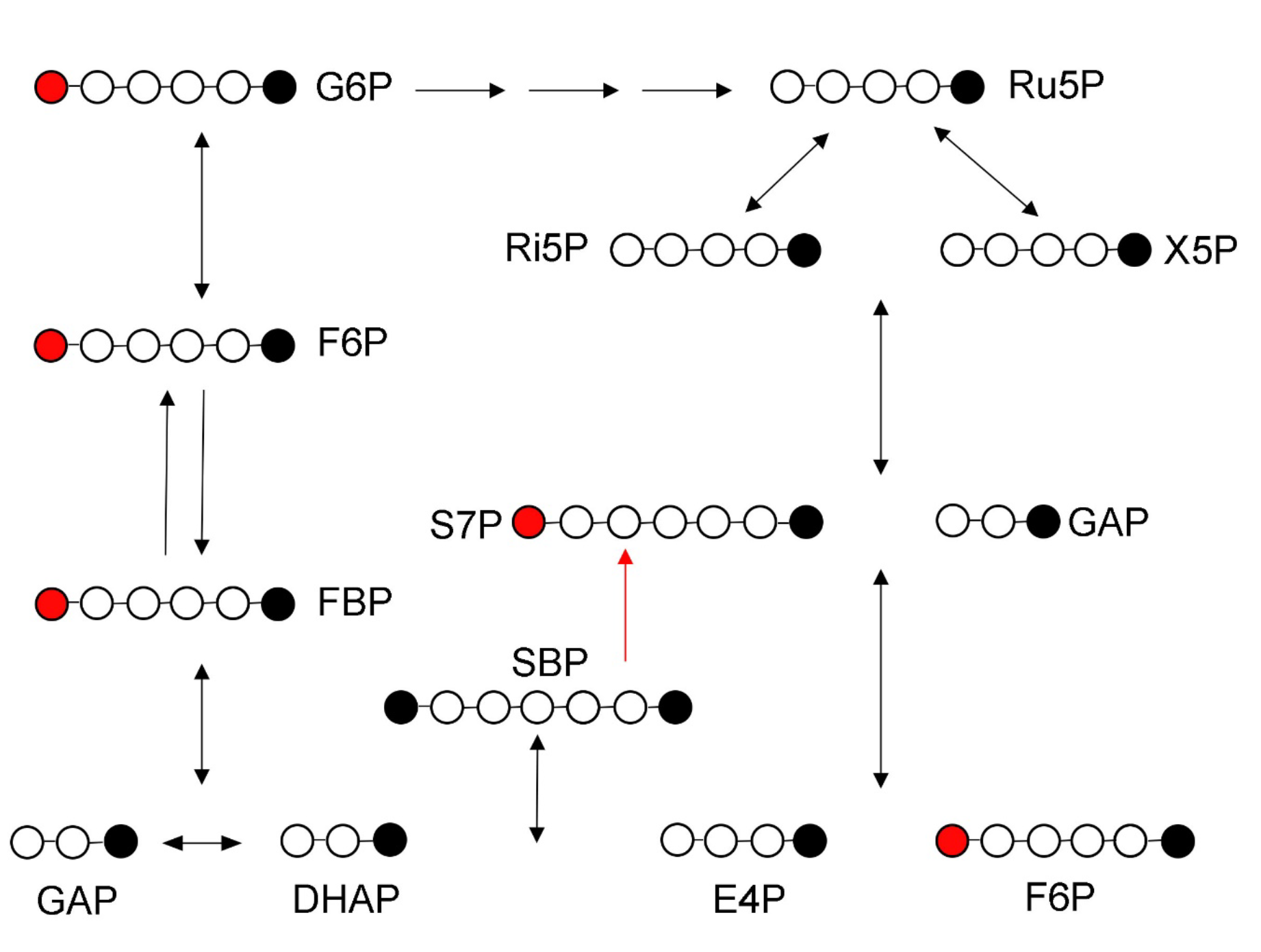

Supplement: S4 Fig — Schematic of the PPP detailed in Fig 4. Simplified diagram labeling assay previously developed (19) shows the potential metabolism of glucose labeled with 13C at the sixth carbon. Each circle is a carbon atom, and all molecules have open circles indicating unlabeled carbons and black circles indicating 13C labeled carbons. Some molecules have red circles, indicating a potential second 13C label. Enzyme catalyzed reactions are indicated with either double or single ended arrows to indicate directionality, with the red arrow indicating the proposed new activity catalyzed by SBPase. Abbreviations are as follows: glucose-6-phosphate (G6P), fructose-6-phosphate (F6P), fructose-1,6-bisphosphate (FBP), glyceraldehyde-3-phosphate (GAP), dihydroxyacetone-phosphate (DHAP), erythrose-4-phosphate (E4P), sedoheptulose-1,7-bisphosphate (SBP), sedoheptulose-7-phosphate (S7P), ribose-5-phosphate (Ri5P), xylulose-5-phosphate (X5P), and ribulose-5-phosphate (Ru5P). (TIF) [file ppat.1008432.s004.tif]

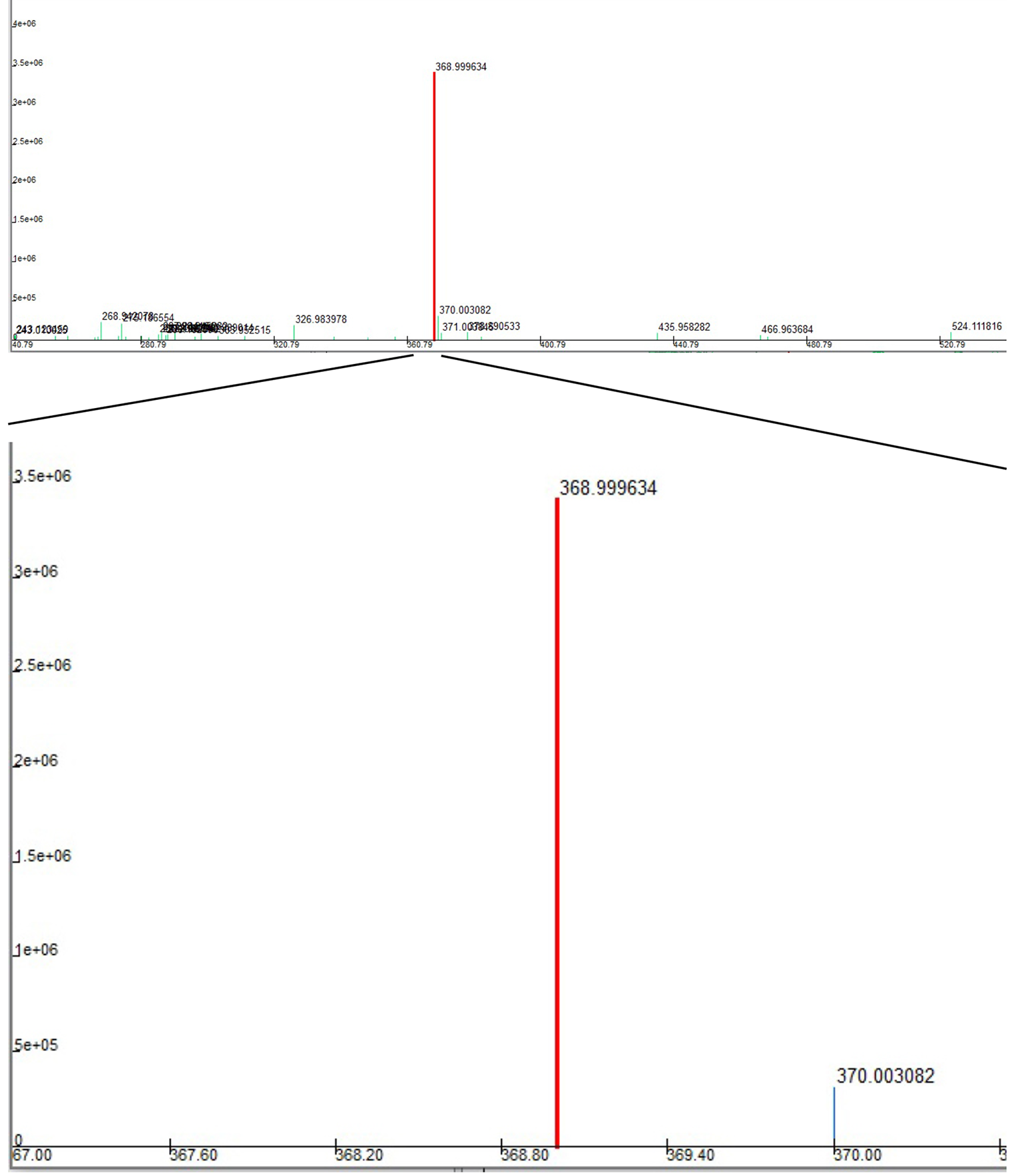

Supplement: S5 Fig — SBP was synthesized by reacting erythrose-4-phosphate and dihydroxyacetone-phosphate with fructose bisphosphate aldolase and product formation was checked by HPLC-MS in negative ionization mode. A dominate peak was seen at 369 kDa, corresponding to the molecular mass of SBP. A minor peak was seen at 370 kDa, likely corresponding to non- or alternatively ionized SBP. The bottom panel is a close-up of these two peaks from the top panel. (TIF) [file ppat.1008432.s005.tif]

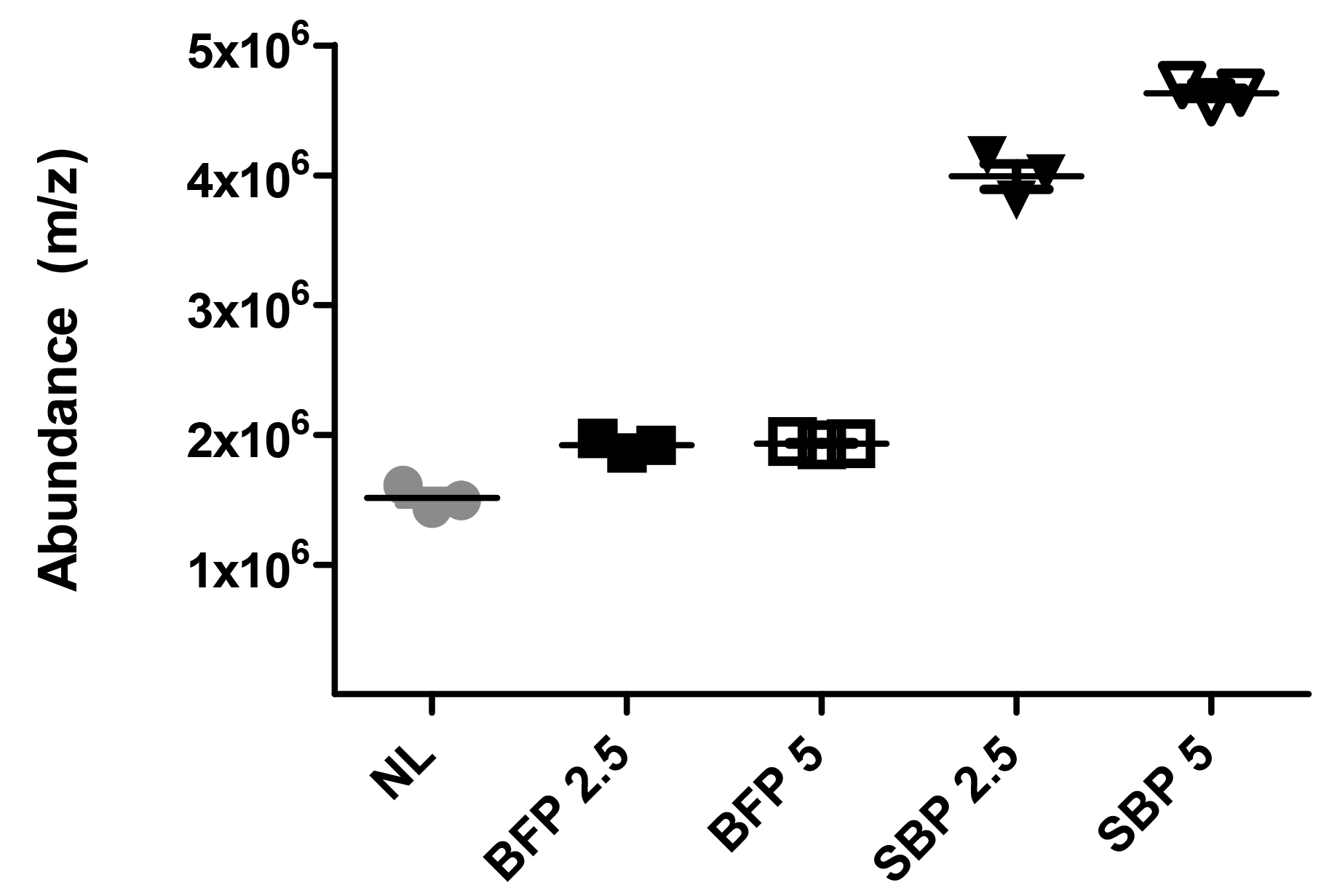

Supplement: S6 Fig — SBP was synthesized from erythrose-4-phosphate, dihydroxyacetone phosphate, and fructose bisphosphate aldolase for 15 minutes. S7P abundance was measured in triplicate after incubation with either no lysate (grey circles), BFP expressing HeLa cell lysate (black shapes), or SBPase expressing HeLa cell lysate (white shapes). Incubation was carried out with either 2.5 μg (squares) or 5 μg (triangles) lysate for 60 minutes. Error bars mark mean and a 95% confidence interval. (TIF) [file ppat.1008432.s006.tif]

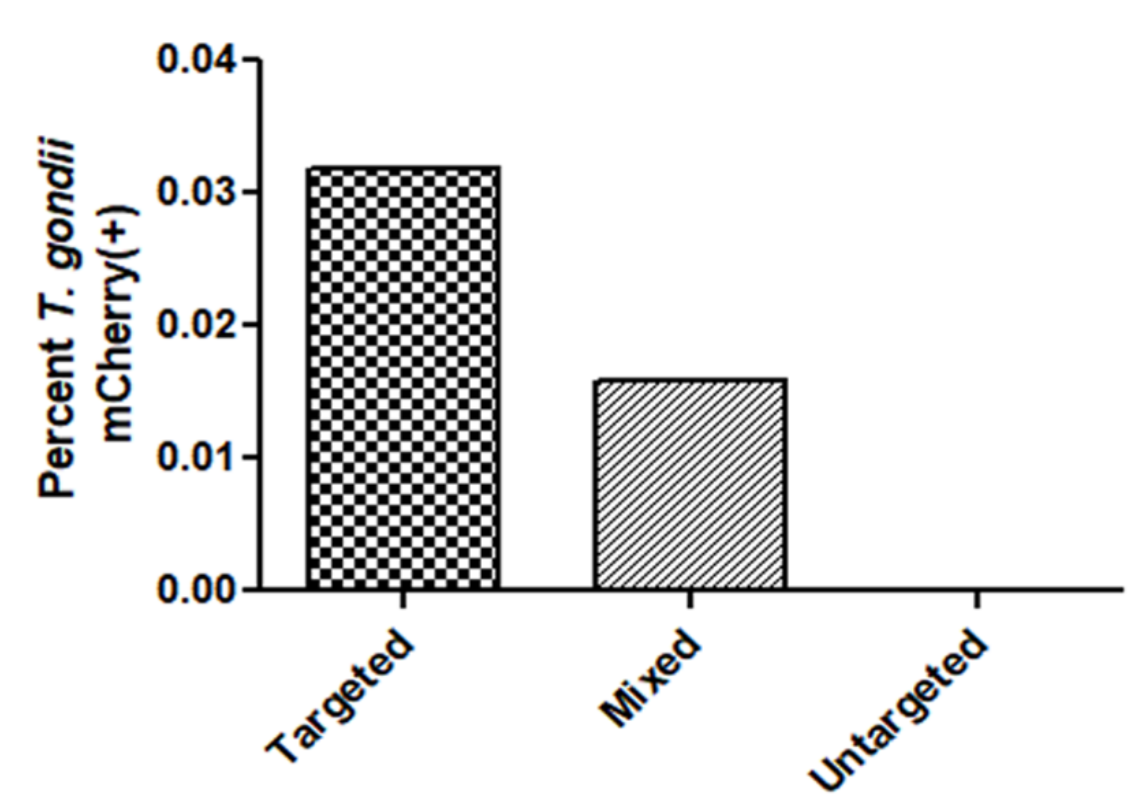

Supplement: S7 Fig — ME49 parasites were electroporated with an mCherry positive selectable marker and one of three CRISPR/cas-9 plasmids; SBPase targeted CRISPR/Cas-9 (Targeted), untargeted CRISPR/Cas-9 (Untargeted), and an equal mix of SBPase and untargeted CRISPR/Cas-9 (Mixed). Electroporated parasites recovered and grew for 72 hours before FACS sorting and cloning. The mixed CRISPR population had half as many mCherry positive parasites as the SBPase targeted population and the untargeted CRISPR population had no mCherry expressing parasites. (TIF) [file ppat.1008432.s007.tif]

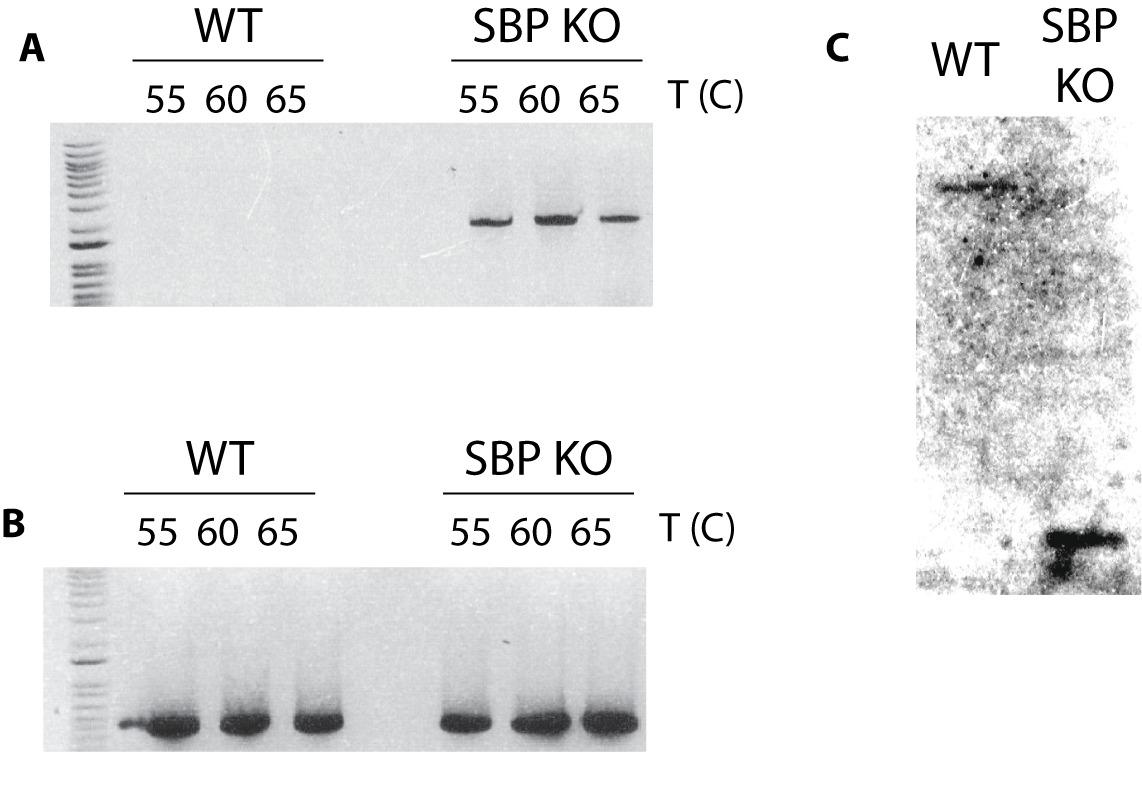

Supplement: S8 Fig — (A) The reaction used mCherry-3’UTR FW and mCherry-3’UTR RV with genomic DNA and reactions were run using a temperature gradient from 55 to 65°C. Expect band size is 2.2 kb only in ΔSBPase. DNA markers in the far-left lane are the 1 kb Plus (Thermo) and the arrowhead shows the 1.5 kb band. (B) SAG-1 FW and SAG1 RV primers used as control of the genomic DNA quality and reactions were run using a temperature gradient from 55 to 65°C. Expect band size is 250 bp in all lanes and DNA markers in the far-left lane are the 1 kb Plus (Thermo) and the arrowhead shows the 1.5 kb band. (C) Genomic DNA from wild type RH (WT) or ΔSBPase parasites was extracted, digested with SacI, separated in an agarose gel, transferred to a positively charged membrane (Amersham), and incubated with 32P-labelled probe specific to the TGME49_235700 downstream region. The arrow indicates the 8 kb band expected for WT parasites; the arrowhead shows the 1.5 kb band expected for ΔSBPase parasites. (TIF) [file ppat.1008432.s008.tif]

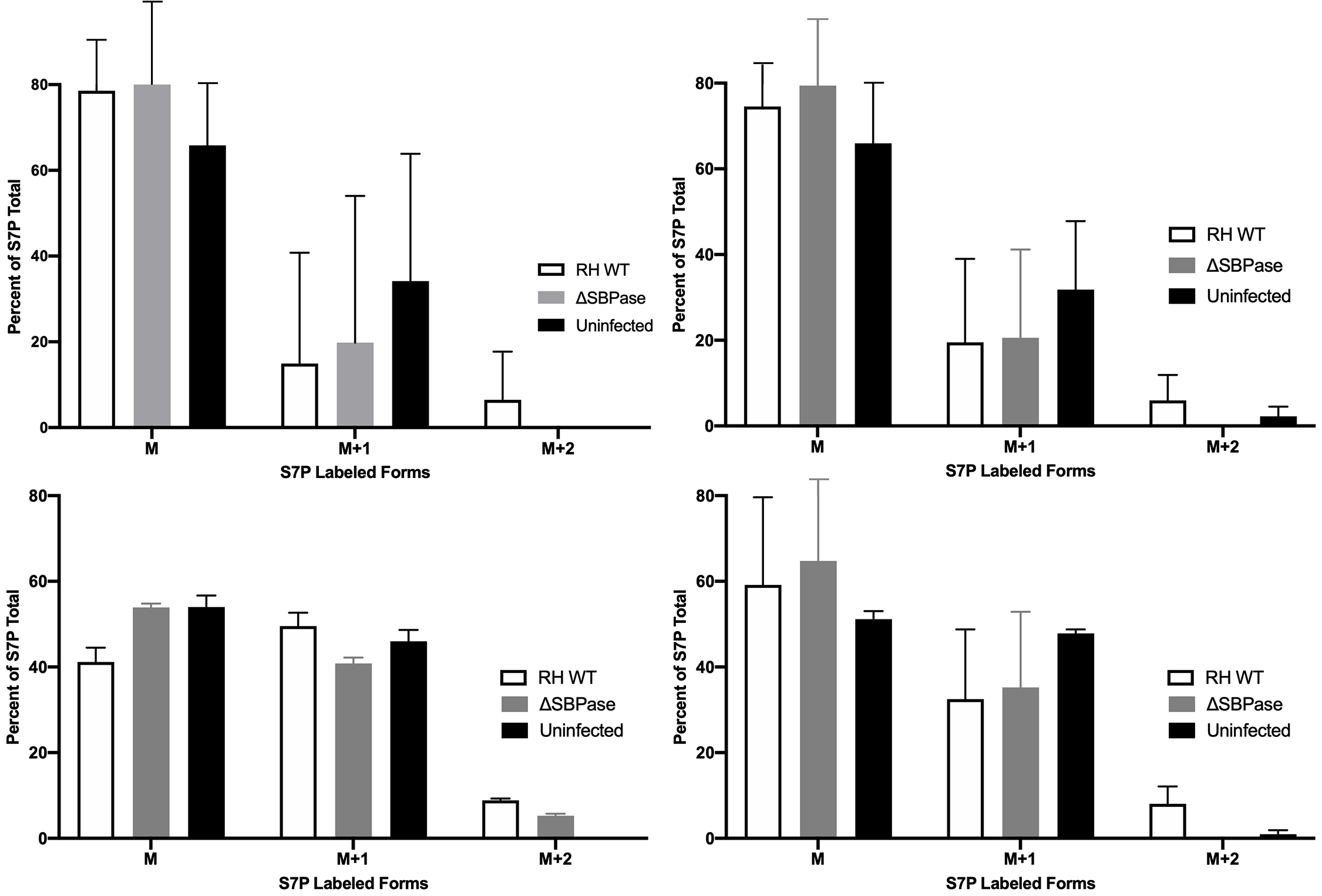

Supplement: S9 Fig — Mass (M) of S7P as a percentage of the total in HFF cells infected with wild type RH (RH WT, white bars) or ΔSBPase parasites (grey bars), or cells left uninfected (black bars). M+1 is S7P containing one 13C and M+2 is S7P containing two 13C. Error bars are a 95% confidence interval. Each of the four independent experiments was performed in triplicate. (TIF) [file ppat.1008432.s009.tif]

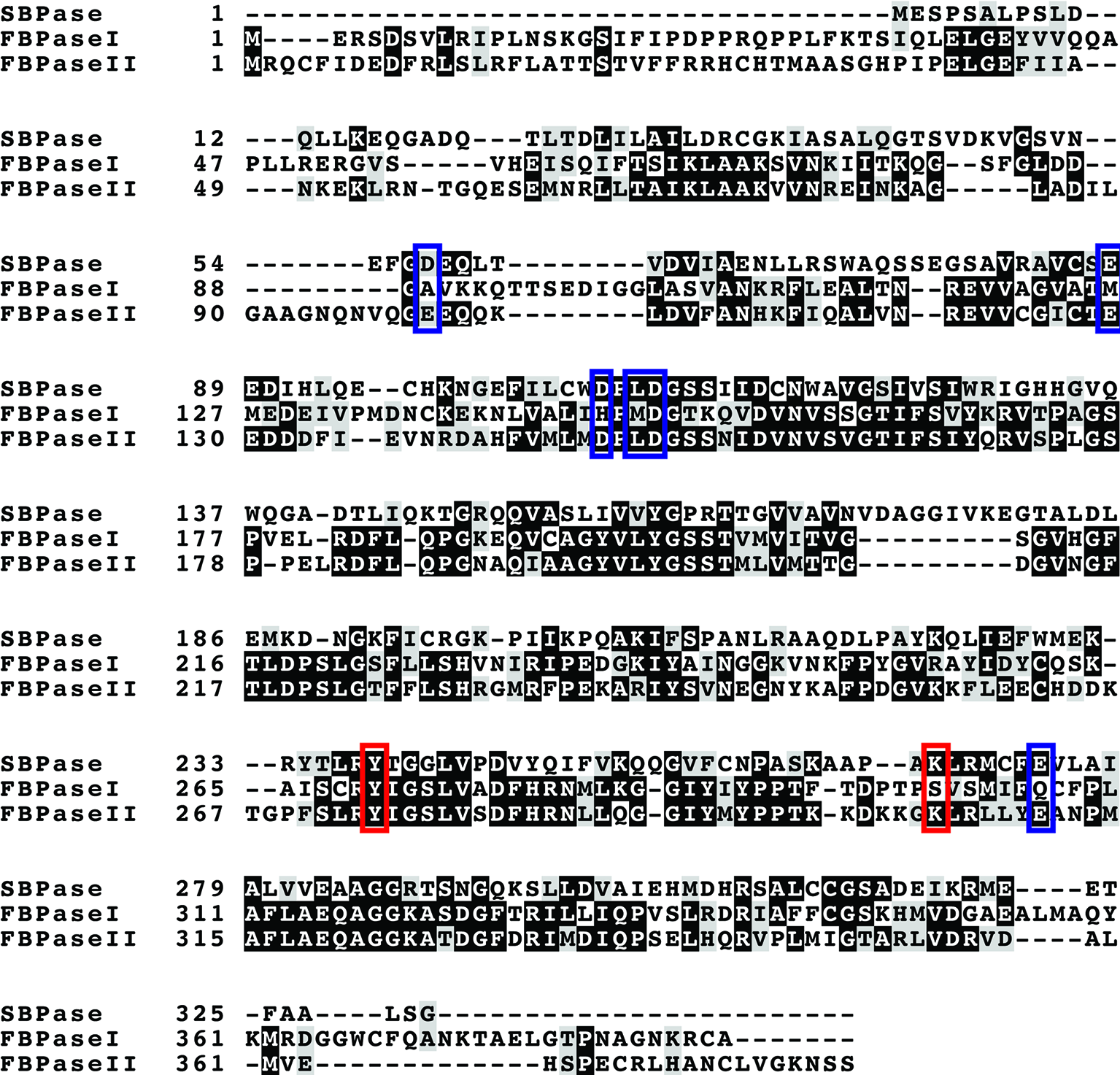

Supplement: S10 Fig — The predicted amino acid sequences from TGME49_235700 (SBPase), TGME49_205380 (FBPase I) and TGME49_247510 FBPase II were aligned using the T-Coffee program [44] and presented using ExPASy BoxShade (https://embnet.vital-it.ch/software/BOX_form.html). Residues of SBP predicted for metal binding are boxed in blue and substrate binding are boxed in red (https://www.uniprot.org/uniprot/P46283). (TIF) [file ppat.1008432.s010.tif]

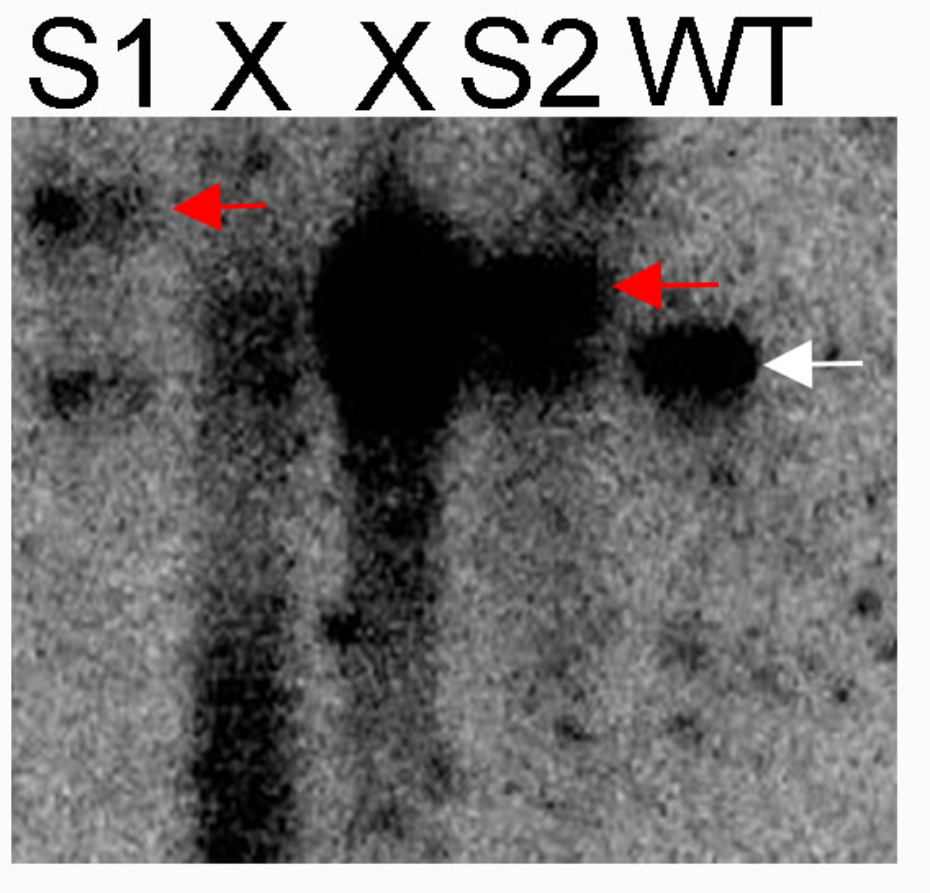

Supplement: S11 Fig — Southern blotting identified two unique SBPOE clones. Genomic DNA was extracted from clonal populations of T. gondii, digested with restriction enzyme XbaI and assayed via Southern blot with an SBPase targeted probe. XbaI digestion yields an expected wild type SBPase band of 5 kb, while the overexpression inserts should yield a second band of unknown length depending on where the overexpression clone was inserted into the genome. A 5 kb WT band was present in all populations and a secondary band was observed in SBPOE1 (S1) and SBPOE2 (S2), indicating a second SBPase gene insertion. The secondary band sizes were different in SBPOE1 and SBPOE2, showing they are unique clones. A white arrow indicates the wild type band in the wild type sample, while two red arrows indicate the secondary bands in the SBPOE clones. Although the clones marked with an X had insertions, they were not used in any further experiments. (TIF) [file ppat.1008432.s011.tif]
