# Supplementary material for: Dual metabolomic profiling uncovers Toxoplasma manipulation of the host metabolome and the discovery of a novel parasite metabolic capability
Source: PLoS Pathog. 2020 Apr 7;16(4):e1008432. doi: 10.1371/journal.ppat.1008432 (PMC7164669; doi:10.1371/journal.ppat.1008432)
Supplement: S1 Table — Metabolite abundance values were compared between triplicate infected and uninfected samples for each metabolite and at each time point over the infection time course using a two tailed t-test. Shown are the p-values of these changes for each metabolite at each time point. (DOCX) [file ppat.1008432.s012.docx]

Hours Post Infection

| Metabolite | 1.5 | 3 | 6 | 9 | 12 | 24 | 36 | 48 |
| --- | --- | --- | --- | --- | --- | --- | --- | --- |
| Serine | 0.08 | 0.83 | 0.29 | 0.16 | 0.65 | 0.01 | 0.00 | 0.12 |
| Threonine | 0.05 | 0.78 | 0.35 | 0.10 | 0.21 | 0.01 | 0.00 | 0.00 |
| Taurine | 0.88 | 0.07 | 0.03 | 0.00 | 0.00 | 0.00 | 0.00 | 0.00 |
| Hydroxyproline | 0.04 | 0.12 | 0.50 | 0.46 | 0.31 | 0.00 | 0.00 | 0.01 |
| Acetylphosphate | 0.14 | 0.03 | 0.06 | 0.04 | 0.02 | 0.04 | 0.17 | 0.10 |
| Glutamine | 0.68 | 0.41 | 0.42 | 0.58 | 0.25 | 0.00 | 0.00 | 0.26 |
| Methionine | 0.02 | 0.54 | 0.51 | 0.78 | 0.46 | 0.09 | 0.07 | 0.78 |
| Tyrosine | 0.24 | 0.85 | 0.55 | 0.59 | 0.47 | 0.30 | 0.30 | 0.51 |
| 3-phosphoserine | 0.41 | 0.24 | 0.17 | 0.01 | 0.03 | 0.05 | 0.01 | 0.05 |
| Tryptophan | 0.05 | 0.08 | 0.50 | 0.76 | 0.37 | 0.01 | 0.01 | 0.13 |
| Argininosuccinate | 0.81 | 0.93 | 0.51 | 0.03 | 0.11 | 0.00 | 0.00 | 0.04 |
| Xanthine | 0.71 | 0.19 | 0.22 | 0.38 | 0.64 | 0.13 | 0.04 | 0.25 |
| Thymidine | 0.30 | 0.28 | 0.22 | 0.08 | 0.18 | 0.13 | 0.06 | 0.16 |
| Uridine | 0.07 | 0.19 | 0.22 | 0.58 | 0.28 | 0.05 | 0.05 | 0.06 |
| Inosine | 0.28 | 0.27 | 0.06 | 0.32 | 0.15 | 0.07 | 0.10 | 0.07 |
| Guanosine | 0.06 | 0.22 | 0.12 | 0.19 | 0.05 | 0.02 | 0.02 | 0.05 |
| 5-phosphoribosyl-1-pyrophosphate | 0.22 | 0.12 | 0.04 | 0.05 | 0.03 | 0.03 | 0.05 | 0.09 |
| CMP | 0.31 | 0.02 | 0.10 | 0.01 | 0.00 | 0.10 | 0.10 | 0.04 |
| UMP | 0.89 | 0.01 | 0.68 | 0.50 | 0.13 | 0.25 | 0.07 | 0.99 |
| CDP | 0.59 | 0.33 | 0.37 | 0.17 | 0.27 | 0.06 | 0.05 | 0.07 |
| GDP | 0.64 | 0.64 | 0.75 | 0.40 | 0.29 | 0.09 | 0.06 | 0.22 |
| dCTP | 0.05 | 0.79 | 0.08 | 0.56 | 0.55 | 0.09 | 0.05 | 0.10 |
| dTTP | 0.04 | 0.05 | 0.00 | 0.05 | 0.17 | 0.03 | 0.01 | 0.03 |
| dATP | 0.03 | 0.06 | 0.00 | 0.02 | 0.13 | 0.04 | 0.00 | 0.01 |
| ATP | 0.79 | 0.98 | 0.07 | 0.51 | 0.08 | 0.97 | 0.13 | 0.00 |
| GTP | 0.62 | 0.51 | 0.73 | 0.65 | 0.15 | 0.10 | 0.04 | 0.83 |
| Pyruvate | 0.52 | 0.35 | 0.03 | 0.26 | 0.68 | 0.05 | 0.00 | 0.06 |
| Lactate | 0.86 | 0.35 | 0.26 | 0.23 | 0.09 | 0.04 | 0.05 | 0.80 |
| Phosphoenolpyruvate | 0.60 | 0.28 | 0.01 | 0.47 | 0.30 | 0.05 | 0.04 | 0.42 |
| 2,3-bisphosphoglycerate | 0.09 | 0.06 | 0.04 | 0.00 | 0.00 | 0.02 | 0.24 | 0.19 |
| fructose-1,6-bisphosphate | 0.21 | 0.10 | 0.30 | 0.91 | 0.19 | 0.04 | 0.06 | 0.06 |
| glucose-6-phosphate | 0.06 | 0.00 | 0.03 | 0.00 | 0.38 | 0.06 | 0.00 | 0.63 |
| fructose-6-phosphate | 0.57 | 0.10 | 0.72 | 0.04 | 0.62 | 0.01 | 0.04 | 0.01 |
| dihydroxyacetone-phosphate | 0.24 | 0.17 | 0.12 | 0.02 | 0.24 | 0.03 | 0.03 | 0.73 |
| glyceraldehdye-3-phosphate | 0.44 | 0.80 | 0.98 | 0.70 | 0.09 | 0.01 | 0.07 | 0.00 |
| 3-phosphoglycerate | 0.96 | 0.40 | 0.13 | 0.18 | 0.12 | 0.10 | 0.19 | 0.07 |
| ribose-5-phosphate | 0.75 | 0.47 | 0.79 | 0.22 | 0.31 | 0.05 | 0.06 | 0.55 |
| ribulose-5-phosphate | 0.79 | 0.54 | 0.65 | 0.06 | 0.53 | 0.04 | 0.03 | 0.04 |
| xylulose-5-phosphate | 0.57 | 0.59 | 0.96 | 0.29 | 0.29 | 0.02 | 0.03 | 0.01 |
| glucono-1,5-lactone-6-phosphate | 0.29 | 0.50 | 0.52 | 0.17 | 0.49 | 0.03 | 0.00 | 0.07 |
| 6-phosphogluconate | 0.59 | 0.43 | 0.89 | 0.08 | 0.30 | 0.03 | 0.00 | 0.07 |
| erythrose-4-P | 0.51 | 0.90 | 0.94 | 0.45 | 0.78 | 0.14 | 0.03 | 0.02 |
| sedoheptulose-7-phosphate | 0.98 | 0.07 | 0.74 | 0.82 | 0.78 | 0.02 | 0.01 | 0.08 |
| sedoheptulose-1,7-bisphosphate | 0.03 | 0.19 | 0.30 | 0.04 | 0.02 | 0.10 | 0.05 | 0.89 |
| octulose-1,8-bisphosphate | 0.12 | 0.02 | 0.02 | 0.00 | 0.02 | 0.12 | 0.02 | 0.01 |
| Fumarate | 0.12 | 0.14 | 0.01 | 0.01 | 0.01 | 0.00 | 0.02 | 0.58 |
| Malate | 0.28 | 0.14 | 0.04 | 0.03 | 0.00 | 0.00 | 0.00 | 0.52 |
| Alphaketoglutarate | 0.02 | 0.00 | 0.00 | 0.01 | 0.00 | 0.01 | 0.01 | 0.03 |
| Aconitate | 0.29 | 0.19 | 0.09 | 0.22 | 0.37 | 0.07 | 0.00 | 0.06 |
| citrate/isocitrate | 0.31 | 0.16 | 0.06 | 0.37 | 0.63 | 0.07 | 0.00 | 0.05 |
| Oxaloacetate | 0.49 | 0.58 | 0.16 | 0.31 | 0.44 | 0.26 | 0.00 | 0.08 |
| acetyl-CoA | 0.07 | 0.16 | 0.52 | 0.12 | 0.17 | 0.11 | 0.05 | 0.67 |
| Succinate | 0.33 | 0.47 | 0.97 | 0.67 | 0.40 | 0.13 | 0.00 | 0.61 |
| succinyl-CoA | 0.30 | 0.49 | 0.06 | 0.43 | 0.03 | 0.41 | 0.02 | 0.84 |
| coenzyme A | 0.07 | 0.04 | 0.51 | 0.06 | 0.00 | 0.03 | 0.09 | 0.67 |
| glutathione disulfide | 0.08 | 0.23 | 0.01 | 0.07 | 0.00 | 0.03 | 0.00 | 0.00 |
