# Supplementary material for: Dual metabolomic profiling uncovers Toxoplasma manipulation of the host metabolome and the discovery of a novel parasite metabolic capability
Source: PLoS Pathog. 2020 Apr 7;16(4):e1008432. doi: 10.1371/journal.ppat.1008432 (PMC7164669; doi:10.1371/journal.ppat.1008432)
Supplement: S2 Table — Ct values for qPCR measuring the expression of SBPase relative to a housekeeping gene. In HeLa cells SBPase and ActB expression was measured for SBPase and BFP expressing cells. In T. gondii SBPase and Tub1A expression was measured for wild type, SBPOE1, and SBPOE2 strains. For all data triplicate biological samples were assayed, with each biological sample run with two technical replicates. (DOCX) [file ppat.1008432.s013.docx]

| Cell Type | Gene | Sample | Ct |
| --- | --- | --- | --- |
| HeLa | ACT | SBOE-1A | 16.17723 |
| HeLa | ACT | SBOE-1B | 15.6975 |
| HeLa | ACT | SBOE-2A | 16.26525 |
| HeLa | ACT | SBOE-2B | 15.56993 |
| HeLa | ACT | SBOE-3A | 15.83994 |
| HeLa | ACT | SBOE-3B | 15.80822 |
| HeLa | ACT | BFP-1A | 13.85979 |
| HeLa | ACT | BFP-1B | 14.43298 |
| HeLa | ACT | BFP-2A | 16.3069 |
| HeLa | ACT | BFP-2B | 16.47485 |
| HeLa | ACT | BFP-3A | 14.71843 |
| HeLa | ACT | BFP-3B | 15.03019 |
| HeLa | ACT | SBOE-1A 1:10 | 19.31338 |
| HeLa | ACT | SBOE-1B 1:10 | 19.739 |
| HeLa | ACT | SBOE-1A 1:100 | 23.46167 |
| HeLa | ACT | SBOE-1B 1:100 | 23.27844 |
| HeLa | ACT | SBOE-1A 1:1000 | 29.16891 |
| HeLa | ACT | SBOE-1B 1:1000 | 26.78812 |
| HeLa | SBP | SBOE-1A | 21.47635 |
| HeLa | SBP | SBOE-1B | 21.43644 |
| HeLa | SBP | SBOE-2A | 20.82235 |
| HeLa | SBP | SBOE-2B | 20.98741 |
| HeLa | SBP | SBOE-3A | 21.30018 |
| HeLa | SBP | SBOE-3B | 21.16433 |
| HeLa | SBP | BFP-1A | Undetermined |
| HeLa | SBP | BFP-1B | 36.93917 |
| HeLa | SBP | BFP-2A | Undetermined |
| HeLa | SBP | BFP-2B | Undetermined |
| HeLa | SBP | BFP-3A | Undetermined |
| HeLa | SBP | BFP-3B | Undetermined |
| HeLa | SBP | SBOE-1A 1:10 | 24.15277 |
| HeLa | SBP | SBOE-1B 1:10 | Undetermined |
| HeLa | SBP | SBOE-1A 1:100 | 29.48122 |
| HeLa | SBP | SBOE-1B 1:100 | 28.27908 |
| HeLa | SBP | SBOE-1A 1:1000 | 33.77965 |
| HeLa | SBP | SBOE-1B 1:1000 | 31.81199 |
| *T. gondii* | TUB | WT-1A | 20.96902 |
| *T. gondii* | TUB | WT-1B | 20.6587 |
| *T. gondii* | TUB | WT-2A | 21.05071 |
| *T. gondii* | TUB | WT-2B | 21.21156 |
| *T. gondii* | TUB | WT-3A | 22.28673 |
| *T. gondii* | TUB | WT-3B | 22.29889 |
| *T. gondii* | TUB | OE6-1A | 18.97004 |
| *T. gondii* | TUB | OE6-1B | 18.86116 |
| *T. gondii* | TUB | OE6-2A | 18.82229 |
| *T. gondii* | TUB | OE6-2B | 18.53777 |
| *T. gondii* | TUB | OE6-3A | 18.67112 |
| *T. gondii* | TUB | OE6-3B | 18.39507 |
| *T. gondii* | TUB | OE9-1A | 20.83246 |
| *T. gondii* | TUB | OE9-1B | 20.89606 |
| *T. gondii* | TUB | OE9-2A | 21.98443 |
| *T. gondii* | TUB | OE9-2B | 21.43806 |
| *T. gondii* | TUB | OE9-3A | 21.79685 |
| *T. gondii* | TUB | OE9-3B | 21.66474 |
| *T. gondii* | TUB | WT-1A 1:10 | 26.67721 |
| *T. gondii* | TUB | WT-1B 1:10 | 26.60336 |
| *T. gondii* | TUB | WT-1A 1:100 | 31.14374 |
| *T. gondii* | TUB | WT-1B 1:100 | 31.03452 |
| *T. gondii* | TUB | WT-1A 1:1000 | 36.95737 |
| *T. gondii* | TUB | WT-1B 1:1000 | 35.04285 |
| *T. gondii* | SBP | WT-1A | 25.43822 |
| *T. gondii* | SBP | WT-1B | 25.24385 |
| *T. gondii* | SBP | WT-2A | 25.68221 |
| *T. gondii* | SBP | WT-2B | 25.48087 |
| *T. gondii* | SBP | WT-3A | 27.19367 |
| *T. gondii* | SBP | WT-3B | 27.19539 |
| *T. gondii* | SBP | OE6-1A | 21.56223 |
| *T. gondii* | SBP | OE6-1B | 21.27877 |
| *T. gondii* | SBP | OE6-2A | 20.94971 |
| *T. gondii* | SBP | OE6-2B | 20.73588 |
| *T. gondii* | SBP | OE6-3A | 20.6483 |
| *T. gondii* | SBP | OE6-3B | 20.54301 |
| *T. gondii* | SBP | OE9-1A | 23.13153 |
| *T. gondii* | SBP | OE9-1B | 23.24938 |
| *T. gondii* | SBP | OE9-2A | 23.51437 |
| *T. gondii* | SBP | OE9-2B | 23.67533 |
| *T. gondii* | SBP | OE9-3A | 22.41254 |
| *T. gondii* | SBP | OE9-3B | 22.0804 |
