# Supplementary material for: Dual metabolomic profiling uncovers Toxoplasma manipulation of the host metabolome and the discovery of a novel parasite metabolic capability
Source: PLoS Pathog. 2020 Apr 7;16(4):e1008432. doi: 10.1371/journal.ppat.1008432 (PMC7164669; doi:10.1371/journal.ppat.1008432)
Supplement: S3 Table — The mean of the fold change of infected over uninfected (Log2) for each metabolite abundance during the infection time course from 1.5 to 48 hours post infection, with each column representing a time point. These means are graphically represented in the large heat map in Fig 1 and the p-values for each are listed in S1 Table. (DOCX) [file ppat.1008432.s014.docx]

| Compound | 1.5 | 3 | 6 | 9 | 12 | 24 | 36 | 48 |
| --- | --- | --- | --- | --- | --- | --- | --- | --- |
| Serine | 0.46 | -0.03 | 0.98 | 0.24 | 0.04 | 0.77 | 0.70 | 0.25 |
| Threonine | 0.47 | 0.05 | 0.60 | 0.28 | 0.17 | 0.65 | 0.72 | 0.53 |
| Taurine | -0.02 | -0.23 | -0.91 | -1.44 | -2.02 | -1.77 | -2.34 | -3.81 |
| Hydroxyproline | 0.42 | -0.16 | 0.42 | 0.13 | 0.09 | 0.87 | 0.69 | 0.35 |
| Acetylphosphate | 0.31 | 0.77 | 0.64 | 1.07 | 0.92 | 0.90 | 0.69 | -0.71 |
| Glutamine | 0.18 | -0.44 | 0.89 | 0.13 | 0.28 | 0.83 | 0.97 | 0.11 |
| Methionine | 0.77 | -0.14 | 0.94 | 0.05 | -0.08 | 0.50 | 0.38 | -0.04 |
| Tyrosine | 0.96 | 0.13 | 0.97 | -0.25 | -0.53 | 0.60 | 0.69 | -0.44 |
| 3-phosphoserine | -0.48 | -0.69 | 0.95 | 1.77 | 1.44 | 3.42 | 1.99 | 1.18 |
| Tryptophan | 0.41 | -0.28 | 0.86 | -0.07 | -0.20 | 0.68 | 0.55 | -0.20 |
| Argininosuccinate | 0.19 | -0.04 | 0.28 | 1.58 | 0.80 | 2.72 | 2.33 | 0.98 |
| Xanthine | -0.17 | 0.70 | 1.73 | 0.34 | 0.19 | 0.80 | 0.99 | 0.33 |
| Thymidine | 0.97 | 0.95 | 3.00 | 1.44 | 0.87 | 3.68 | 4.12 | 4.14 |
| Uridine | 1.05 | 0.86 | 0.55 | 0.32 | 0.53 | 1.81 | 1.91 | 1.76 |
| Inosine | 0.78 | 1.03 | 0.94 | 0.82 | 1.10 | 1.88 | 1.73 | 1.92 |
| Guanosine | 0.89 | 1.04 | 1.09 | 1.17 | 1.30 | 2.48 | 3.00 | 3.56 |
| 5-phosphoribosyl-1-pyrophosphate | 0.51 | 0.77 | 1.08 | 1.35 | 0.91 | 1.57 | 2.01 | 0.87 |
| CMP | 0.69 | 0.71 | 0.87 | 0.86 | 1.42 | 2.39 | 3.49 | 2.52 |
| UMP | -0.05 | 0.53 | 0.12 | 0.19 | 0.58 | 1.01 | 2.36 | 0.00 |
| CDP | 0.39 | 0.67 | 0.27 | 0.71 | 0.88 | 1.25 | 2.22 | 0.97 |
| GDP | -0.35 | 0.27 | -0.11 | 0.46 | 0.50 | 0.80 | 1.51 | 0.54 |
| dCTP | 0.38 | 0.06 | -0.25 | -0.18 | -0.14 | 1.13 | 1.37 | 0.50 |
| dTTP | 1.21 | 0.82 | 0.80 | 0.73 | 0.53 | 2.40 | 2.70 | 2.03 |
| dATP | 1.42 | 0.93 | 0.72 | 0.81 | 0.41 | 2.02 | 2.21 | 1.50 |
| ATP | 0.04 | 0.00 | -0.23 | -0.12 | -0.20 | 0.00 | -0.40 | -1.54 |
| GTP | 0.14 | 0.23 | -0.05 | 0.16 | 0.39 | 1.06 | 1.17 | -0.06 |
| Pyruvate | 0.32 | 0.40 | 0.78 | 0.30 | 0.10 | 1.17 | 2.01 | 1.68 |
| Lactate | 0.03 | 0.19 | 0.66 | 0.13 | -0.42 | 1.11 | 0.96 | -0.09 |
| Phosphoenolpyruvate | -0.21 | 1.01 | 0.87 | 0.54 | 0.64 | 1.93 | 1.50 | 0.31 |
| 2,3-bisphosphoglycerate | 0.88 | 0.68 | 1.45 | 2.24 | 2.25 | 4.06 | 1.41 | -0.84 |
| fructose-1,6-bisphosphate | 0.34 | -0.39 | -0.31 | 0.03 | 0.45 | 1.20 | 0.87 | -0.62 |
| glucose-6-phosphate | -0.51 | -0.98 | -0.76 | -0.68 | -0.22 | 0.74 | 0.92 | 0.13 |
| fructose-6-phosphate | -0.18 | -0.29 | 0.11 | 0.24 | 0.10 | 0.77 | 0.50 | -0.70 |
| dihydroxyacetone-phosphate | 0.58 | 0.56 | 0.89 | 1.17 | 0.59 | 1.46 | 1.08 | 0.12 |
| glyceraldehdye-3-phosphate | 0.20 | -0.05 | -0.01 | -0.15 | 0.35 | 1.44 | 0.43 | -1.43 |
| 3-phosphoglycerate | -0.03 | 0.37 | 0.48 | 0.83 | 0.84 | 0.86 | 0.66 | -0.71 |
| ribose-5-phosphate | 0.16 | -0.26 | 0.09 | 0.39 | 0.20 | 2.01 | 1.24 | -0.39 |
| ribulose-5-phosphate | 0.07 | -0.13 | -0.11 | 0.36 | 0.08 | 1.26 | 0.58 | -0.72 |
| xylulose-5-phosphate | -0.09 | 0.12 | 0.04 | 0.32 | 0.24 | 2.86 | 3.94 | 3.52 |
| glucono-1,5-lactone-6-phosphate | 1.07 | -0.26 | -0.24 | 0.82 | 0.54 | 3.47 | 4.92 | 4.07 |
| 6-phosphogluconate | -0.25 | -0.23 | -0.03 | 0.60 | 0.41 | 2.57 | 3.86 | 3.34 |
| erythrose-4-P | 0.83 | 0.04 | -0.08 | 0.58 | -0.13 | 4.16 | 4.36 | 3.29 |
| sedoheptulose-7-phosphate | 0.00 | -0.27 | -0.14 | -0.03 | -0.07 | 1.65 | 2.26 | 1.26 |
| sedoheptulose-1,7-bisphosphate | 0.65 | 0.24 | 0.30 | 0.65 | 1.01 | 1.55 | 0.81 | 0.03 |
| octulose-1,8-bisphosphate | 0.95 | 0.84 | 1.30 | 2.09 | 1.85 | 2.98 | 1.37 | 1.85 |
| Fumarate | 0.51 | 0.41 | 0.44 | 0.56 | 0.56 | 1.20 | 1.12 | 0.10 |
| Malate | 0.38 | 0.44 | 0.80 | 0.88 | 0.62 | 1.65 | 1.29 | -0.10 |
| Alphaketoglutarate | 1.48 | 0.88 | 1.59 | 1.21 | 1.05 | 2.55 | 2.37 | 1.08 |
| Aconitate | 0.53 | 0.65 | 0.72 | 0.60 | 0.49 | 1.31 | 1.93 | 1.43 |
| citrate/isocitrate | 0.56 | 0.62 | 0.97 | 0.36 | 0.22 | 1.53 | 2.37 | 2.00 |
| Oxaloacetate | -0.51 | -0.34 | 0.30 | 0.93 | -0.42 | 1.43 | 2.74 | 2.69 |
| acetyl-CoA | 0.33 | 0.24 | 0.07 | 0.37 | 0.15 | 0.95 | 1.11 | 0.13 |
| Succinate | -0.24 | 0.32 | -0.01 | 0.04 | -0.16 | 0.33 | 0.77 | -0.33 |
| succinyl-CoA | 0.28 | 0.31 | 0.44 | 0.33 | 0.54 | 0.67 | 0.92 | -0.09 |
| coenzyme A | 0.60 | 0.61 | 0.16 | 0.43 | 0.70 | 1.21 | 0.97 | -0.13 |
| glutathione disulfide | -0.78 | -0.70 | -1.33 | -0.93 | -1.62 | -1.07 | -1.28 | -2.07 |
