# Supplementary material for: Dual metabolomic profiling uncovers Toxoplasma manipulation of the host metabolome and the discovery of a novel parasite metabolic capability
Source: PLoS Pathog. 2020 Apr 7;16(4):e1008432. doi: 10.1371/journal.ppat.1008432 (PMC7164669; doi:10.1371/journal.ppat.1008432)
Supplement: S4 Table — All primers are listed in 5’ to 3’ orientation, with the gene they were targeted to and the where experiment they were used. (DOCX) [file ppat.1008432.s015.docx]

| Name | Use | Sequence (5’ to 3’) |
| --- | --- | --- |
| Tub1A Promoter-Fw | SBPase OE in *T. gondii* | Ggcgaattgggtaccgggcctgcatgtccgcgttcgtg |
| Tub1A Promoter-Rv | SBPase OE in *T. gondii* | tttgggaggacattttgtcgaaaaagggaattcaagaaaaaatg |
| SBPOE-Fw | SBPase OE in *T. gondii* | Cgacaaaatgtcctcccaaagcagagaaaatg |
| SBPOE-Rv | SBPase OE in *T. gondii* | Ccactagggccgctctagaaccatgaagtcccagcggg |
| SBP-Fw | SBPase expression in HeLa | Caccatggagtcgccttctgc |
| SBP-Rv | SBPase expression in HeLa | Ctagccggagagtgcagcaaac |
| KO US-FW | SBPase knockout | gctccaattgacatatggatctactcggatctcctctttc |
| KO US-RV | SBPase knockout | gccatcaagctgctagcgataggctcattcttttgcatgc |
| KO DS-FW | SBPase knockout | ccaccaacactgcagaatttgcgttccaaactgacgttga |
| KO DS-RV | SBPase knockout | ggtcttaaggcatgctatttgacaaacccgattttttaaa |
| KO GFP-Fw | SBPase knockout | agcatgccttaagacctaggatgtccgcgttcgtgaaattc |
| KO GFP-Rv | SBPase knockout | aatcgagacgccacagatcttcgggggggcaagaattg |
| KO mCherry-Fw | SBPase knockout | atgagcctatcgctagtgtcccgcgttcgtgaaattc |
| KO mCherry-Rv | SBPase knockout | gtgttggtggcggccgtgtcactgtagcctgccagaac |
| Cas9 FW | SBPase gRNA | AAATGAATCAGTTTTAGAGCTAGAAATAGC |
| Cas9 RV | SBPase gRNA | TCTCTGCTTTAACTTGACATCCCCATTTAC |
| qSBP-Fw | qPCR of SBPase | acagacctcatcttggctattc |
| qSBP-Rv | qPCR of SBPase | gttcgtcgccgaattcattg |
| qTub1A-Fw | qPCR of *T. gondii* Tub1A | gacgacgccttcaacaccttcttt |
| qTub1A-Rv | qPCR of *T. gondii* Tub1A | agttgttcgcagcatcctctttc |
| qActB-FW | qPCR of human ActB | cgtactcctctgcattgtctc |
| qActB-RV | qPCR of human ActB | agcactgtgttggcgtacag |
| SBPOE South-FW | Southern blotting | tctgtgctgcggaagcg |
| SBPOE South-RV | Southern blotting | cttgccaagcacacgaacg |
| SAG-1 FW | PCR of *T. gondii* SAG-1 | TGCCCAGCGGGTACTACAAG |
| SAG-1 RV | PCR of *T. gondii* SAG-1 | TGCCGTGTCGAGACTAGCAG |
| KO Probe FW | Southern blotting | ACGTTCCCGTCTTGTCAAAG |
| KO Probe RV | Southern blotting | ATCTCGAAACGGTGTGGAAG |
| mCherry-3 UTR FW | PCR of mCherry and SBPase 3’UTR | CCCCTGTGTCCTTTATCGAA |
| mCherry-3 UTR RV | PCR of mCherry and SBPase 3’UTR | GACTAGCGCAAGCAGGAACT |
